# Comparing the net-energy balance of standalone photovoltaic-coupled electrolysis and photoelectrochemical hydrogen production†

**DOI:** 10.1039/d3ee02814c

**Published:** 2024-01-15

**Authors:** Brian Tam, Oytun Babacan, Andreas Kafizas, Jenny Nelson

**Affiliations:** a Department of Physics, Imperial College London South Kensington London SW7 2AZ UK b.tam18@imperial.ac.uk jenny.nelson@imperial.ac.uk; b Department of Chemistry, Molecular Sciences Research Hub, Imperial College London White City London W12 0BZ UK; c Grantham Institute – Climate Change and the Environment, Imperial College London South Kensington London SW7 2AZ UK

## Abstract

Photovoltaic-coupled electrolysis (PV-E) and photoelectrochemical (PEC) water splitting are two options for storing solar energy as hydrogen. Understanding the requirements for achieving a positive energy balance over the lifetime of facilities using these technologies is important for ensuring sustainability. While neither technology has yet reached full commercialisation, they are also at very different technology readiness levels and scales of development. Here, we model the energy balance of standalone large-scale facilities to evaluate their energy return on energy invested (ERoEI) over time and energy payback time (EPBT). We find that for average input parameters based on present commercialised modules, a PV-E facility shows an EPBT of 6.2 years and ERoEI after 20 years of 2.1, which rises to approximately 3.7 with an EPBT of 2.7 years for favourable parameters using the best metrics amongst large-scale modules. The energy balance of PV-E facilities is influenced most strongly by the upfront embodied energy costs of the photovoltaic component. In contrast, the simulated ERoEI for a PEC facility made with earth abundant materials only peaks at 0.42 after 11 years and about 0.71 after 20 years for facilities with higher-performance active materials. Doubling the conversion efficiency to 10% and halving the degradation rate to 2% for a 10-year device lifetime can allow PEC facilities to achieve an ERoEI after 20 years of 2.1 for optimistic future parameters. We also estimate that recycling the materials used in hydrogen production technologies improves the energy balance by 28% and 14% for favourable-case PV-E and PEC water splitting facilities, respectively.

## Abstract 2

Photovoltaic-coupled electrolysis and photoelectrochemical water splitting are two options for storing solar energy as hydrogen. For each technology, the energy inputs and hydrogen output need to be considered to compare the overall energy balance.

## Graphical abstract

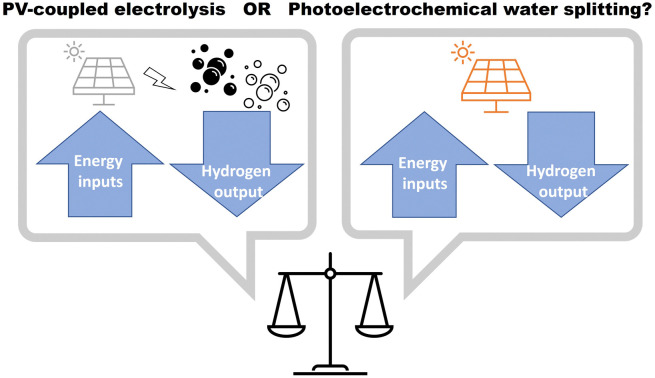

**Broader context**

Hydrogen gas is regarded as a key energy vector for decarbonising transportation and industry. One way of producing green hydrogen from water with very low carbon emissions is with direct solar-driven water splitting. An already mature technology for solar-driven water splitting is photovoltaic-coupled electrolysis (PV-E), while photoelectrochemical (PEC) water splitting has attracted significant research effort due to its potential for simplicity and cost savings. Neither technology, however, is as yet implemented widely and research is still ongoing to improve both technologies. An energy balance comparison of these two technologies is currently lacking in the literature, despite this comparison being imperative for understanding what technological advances are needed to realise hydrogen as a solar energy storage medium. In this study, we simulate the energy balance over time for PV-coupled electrolyser and PEC water splitting facilities. The energy return on energy invested for most cases of PV-coupled electrolysers is already superior to conventional hydrogen production by steam methane reforming. In contrast, PEC water splitting is at a much earlier stage of technology readiness with its low efficiency and relatively fast degradation. We simulate what developments are needed for PEC facilities to provide a positive energy output and find that PEC still struggles to meet the ERoEI of PV-E when using parameters representing future improvements. For both technologies, we find that recycling is one approach to improve the energy balance and limit the extraction of scarce resources. These findings may guide research and development of solar-driven water splitting technologies for green hydrogen production and thereby help to facilitate a future circular economy that incorporates hydrogen as an energy vector.

## 1. Introduction

A low-carbon emissions society requires “green hydrogen”^1^ production, which commonly refers to hydrogen gas (H_2_) produced solely with renewable energy sources. Green H_2_ may stabilise seasonal supply in future renewable energy distribution systems.^2,3^ Green H_2_ may also be targeted to produce “e-fuels” – carbon fuels and high value chemicals made using renewable hydrogen and feedstocks^4^ – that reduce the net-emissions of difficult to electrify sectors such as heavy transportation.^5^ Over 20 million tonnes of additional H_2_ per year by 2050 may be needed as feedstock for primary production of ammonia and methanol.^6^ Existing hydrogen demand for industry (market share of 174 billion USD in 2022^5^)^1^ included 48 million tonnes of H_2_ in 2019 as a by-product of fossil fuel extraction and required an additional 70 million tonnes to be produced by steam methane reforming (SMR) or coal gasification that year.^6^ Every 1 kg of H_2_ produced by SMR results in approximately 10 kg of direct carbon dioxide (CO_2_) emissions.^6^ Therefore, swiftly implementing green hydrogen production technologies is vital for both facilitating a low-emissions energy system and reducing greenhouse gas emissions from H_2_ production (globally 900 million tonnes of CO_2_ in 2020 and 2.5% of energy and industry related emissions^7^).

Photovoltaic-coupled electrolysis (PV-E)^8^ and photoelectrochemical (PEC) water splitting^9^ are two promising methods of solar-driven H_2_ production,^10–12^ where hydrogen is produced by extracting hydrogen from water (“water splitting”) using solar energy. Amongst state-of-the-art laboratory-scale devices, the best systems use inorganic semiconductors^13^ and have been demonstrated with solar-to-hydrogen (STH) efficiencies over 30% for PV-E^14^ and 19% STH for PEC devices.^15^ While PV-E technology is already mature with a technology readiness level of 9, the long-term viability of PEC water splitting is unclear because of uncertainty over future performance, scalability, and competition with PV-E investment.^16–18^

An important consideration for evaluating options for water splitting is the net energy balance. The efficiency with which a technology provides useful energy for society is often quantified in the literature by the energy return on energy invested (ERoEI or sometimes expressed as EROI). ERoEI is the ratio of the energy output to energy input for an energy producing process. Energy payback time (EPBT) is a related metric of the time for the total energy output to be equal to the energy input costs, when ERoEI is equal to one. The ERoEI and EPBT of PV electricity generation has been intensely studied. One 2017 meta-assessment of studies of mono and polycrystalline silicon cells found that average EPBT halved after 2008 from 3.9 to 2.0 years and average ERoEI doubled from 7 to 14.4 after 2008.^19^ These improvements were found to obey a log-linear learning curve trend due to improvements in PV manufacturing.^20^ The ERoEI values for PV electricity are already competitive with the ERoEI of electricity derived from fossil fuels,^21^ but there are few other energy balance assessments of hydrogen production systems. Examples of ERoEI for green hydrogen production facilities include a study by Yadav *et al.* who reported an ERoEI of 4 for a 30-year lifetime PV and 10-year lifetime alkaline electrolyser (AE),^22^ and a study by Sathre *et al.* who predicted an ERoEI between 2 and 3 for low- to high-cost theoretical PEC water splitting facilities.^23^ Some life cycle analyses^24–27^ do report the energy costs of components but focus their analyses on other outcomes such as environmental impacts and emissions. While energy balance has been analysed individually for photovoltaics, electrolysers, and photoelectrochemical components,^28–31^ there are few analyses on the energy balance of integrated hydrogen production systems.^22,23^ The greenhouse gas equivalent emissions^32,33^ and the levelized cost of hydrogen^34–39^ are more commonly reported than energy cost.

Here, we use a systems-level modelling approach to compare PV-E and PEC facilities with a goal to provide evidence for the energy balance viability of large-scale solar-driven green hydrogen production. The aims of the study are to provide a thorough comparison of the ERoEI and EPBT for PV-E and PEC technologies so that researchers have an indication of the status of the field and the system priorities for improvement in both technologies. We simulate water splitting facilities that limit the use of precious metals or rare elements to represent the most scalable solutions. The PV-E model facility is based on crystalline silicon PV and alkaline electrolyser systems for which pilot plants are already in operation.^40^ For PEC water splitting, the model system is a wired metal-oxide thin film photoelectrode panel^41^ commonly used in prototype demonstrators.^42–45^ Following a description of the simulation methodology and input parameters, we first simulate the dynamic energy balance for the model PV-E and PEC facilities to determine ERoEI over time and the EPBT for different facility parameters and then consider how materials recycling improves energy balance. The simulation is modified for the energy costs of recycling end-of-life components and the energy savings of manufacturing new components using recycled material. The approach taken in this work may be applied to other energy conversion processes. Analysing technologies through the lens of energy recovery is important for informing and potentially directing clean energy research efforts.

## 2. Simulation methodology

We simulate the energy balance of both PV-E and PEC water splitting using system-level models that track several rate equations describing the system performance over time. These models were implemented in Vensim PLE+ software^46^ for ease of system dynamics modelling and high-volume Monte Carlo sensitivity simulations, although models could be implemented with common spreadsheet programs or simple code. Fundamentally, the calculations central to this work involve dividing the total hydrogen energy output from a facility by the total energy input costs at a given time. The energy output is derived from conversion efficiency of solar energy to hydrogen and the energy input costs are a sum of the energy required in the manufacturing, operation and decommissioning of facilities. The ERoEI is calculated at the end of each year of operation and the EPBT is extracted from the time at which ERoEI reaches unity.

Specific assumptions and methodology choices are as follows. The input energy metrics are obtained from literature that typically report the thermal energy equivalents, sometimes alongside electrical units for energy. Electrical output energy such as the electricity from solar PV modules are converted to thermal units by the electrolyser conversion efficiency in the model, so final comparison with the thermal energy input parameters is appropriate to calculate ERoEI. For simplicity and comparability, the systems studied here are standalone without battery storage or grid-connection. Although we do not perform life cycle analyses of the technologies we study, we collect and use results of life cycle and energy balance analysis studies already in the literature. We determine the net energy balance for sets of input parameters and then analyse the sensitivity of the ERoEI and EPBT to changes in factors such as embodied energy costs, maintenance energy costs, efficiency, and degradation rates, among others.

### 2.1. Calculating the energy balance for the PV-E and PEC facilities

**Fig. 1:**
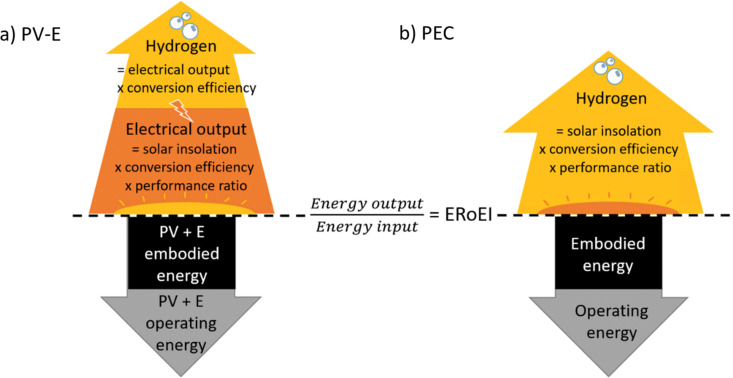
Schematic of the parameters for calculating the ERoEI over time for (a) a PV-E facility and (b) a PEC facility. ERoEI is the ratio of calculations above and below the line.

In the schematics in Fig. 1, the parameters involved in calculating the total hydrogen energy output and total energy input costs are depicted above and below the central dividing line respectively. Briefly, the energy output is derived from solar insolation which is multiplied by a conversion efficiency and performance ratio. In the case of the PV-E facility, this electrical output is once again multiplied by a conversion efficiency to obtain the final hydrogen output energy. The energy input for both technologies is made up of a one-time embodied energy including construction and decommissioning energy costs, and then an operation or maintenance energy cost is added annually. Fig. 1a is a schematic of the energy accounting to calculate the PV-E ERoEI. The energy input cost initially consists of the embodied construction and decommissioning energy costs for the PV and electrolyser module and then grows at a constant rate (the operating energy). The energy output accumulates as solar energy is transformed to electrical energy before being stored as hydrogen (each process includes losses and a diminishing efficiency over time). Fig. S1 (ESI†) shows the working Vensim model for the PV-E system.

Fig. 1b is a schematic of the parameters for calculating the ERoEI of the PEC facility. The energy input cost again initially consists of the embodied construction and decommissioning energy costs and then grows at a constant rate (the operating energy). The energy output accumulates as solar energy is transformed into hydrogen, including losses and a diminishing efficiency over time. Fig. S2 (ESI†) shows the working model for the PEC water splitting facility modelled in Vensim.

#### Calculating ERoEI of the PV-E facility over time

ERoEI at any year, *n*, after the start of building a facility, may be specified as eqn (1) and is the ratio of the cumulative energy contained in the hydrogen gas produced by the facility per meter squared of PV from time 0 to the end of year *n*, 
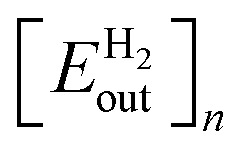
, to the cumulative input energy cost of the facility from time 0 to the end of year n per meter squared of the PV, 
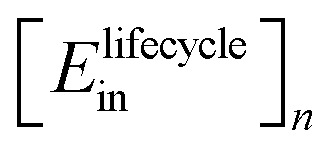
. A full derivation of the calculations for the ERoEI of a PV-E facility may be found in Section S1 of the ESI.†1
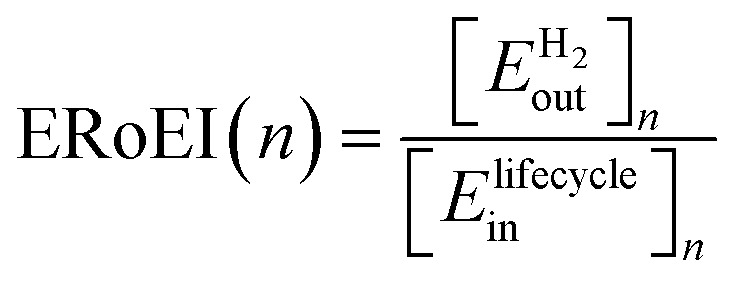


The energy output is the total energy of the hydrogen produced from the electrolyser per meter squared of PV and pressurized to 200 bars (20 MPa) at the plant gate.^28^ Pressurised hydrogen is the typical form for storage or transportation.^47^ All the energy used to operate the electrolyser is assumed to be from electrical energy produced by the PV facility.

While solar photovoltaics and alkaline electrolysers are individually technologically mature, operation of the combined PV-AE technology has additional complications including gas crossover between the cathode and anode that create flammable mixtures and lower conversion efficiencies at low current loads.^48^ To reduce gas crossover, approaches such as improved anion exchange membranes^49^ and system pressure control strategies^50^ exist in the literature. To improve the conversion efficiency at low current loads, Xia *et al.* show that physical structure and electrical characteristics of AE have a large influence on low-load performance and that, for instance, the use of optimised alternating current can extend the operation range where conversion efficiency is greater than 50% down to 10% of the rated load.^51^ Additionally, some studies claim that electrolysers are ramp-rate constrained,^52,53^ but other reports and studies have stated that AE can ramp up and down within seconds.^47,54,55^ Although a 2009 study by Ursua *et al.* showed that high current loads (120 A) may experience up to 3% better efficiency than low current loads (40 A),^56^ advances in technology since then would likely limit efficiency losses. We therefore assume for simplicity that the AE electricity-to-H_2_ conversion efficiency (electrical energy to thermal energy in hydrogen as the lower heating value) is independent of the electrical current received by the AE. The effect of this assumption would be to increase the energy converted by the electrolyser, but the differences primarily affect the low current load regime, which produce a minor portion of the total energy output and which will be mitigated by updated technologies. Still, if applying this model to regions where solar insolation is highly variable over the course of a day, empirical data would be useful to help inform the appropriate average efficiency input parameter.

Here we define the parameters considered in the models and briefly justify our parameter choices in Section 2.2 below. A full discussion of the parameter choices for the PV-E facility may be found in Section S3 of the ESI.† Conversion efficiencies taken from the literature are used here only as initial values that decrease over the lifetime of the facility. In the literature, constant degradation rates over the operational lifetime of a facility are reported^57^ or assumed.^58,59^ It is noteworthy, though, that the cycling of the electrolyser when solely powered by solar energy may increase the rate of degradation over time.^60^ We do not, however, consider this effect here. The performance ratio of PV modules is described by The International Standard IEC 61724 as the electricity generated as alternating current (AC) compared to the PV module's expected direct current (DC) performance^61^ (essentially losses at the DC to AC inverters). For instance, a PV-AE facility with grid connection would need to consider these losses as an upper bound for the performance ratio. A PV module solely coupled to an electrolyser would not strictly need to use a DC–AC converter but would still need a DC–DC converter to ensure the optimal voltage is supplied.^62^ The performance ratio further includes losses due to environmental factors such as soiling, weather, and temperature fluctuations.^27,61^ The upfront energy costs for the PV and AE consider both the embodied energy cost in constructing the facilities and expected decommissioning energy cost associated with landfilling the facilities at its end-of-life. Finally, the maintenance energy costs for the PV consider the replacement of faulty equipment such as modules and inverters that break before the expected lifetime of the PV panels. The operating energy cost to the AE is considered largely to be the energy cost for hydrogen compression to 200 Bar.

#### Calculating electricity ERoEI of a PV facility over time

Similarly to the ERoEI of the PV-E facility, an ERoEI for a PV facility alone may be calculated for electricity output divided by thermal energy inputs. This ratio may be converted to consistent electrical units by multiplying the thermal energy denominator by an efficiency ratio of 0.38, as in Raugei *et al.*,^63^ which is also within the range of 0.3 to 0.7 investigated by Murphy *et al.* in their review of the ERoEI literature.^64^

#### Calculating ERoEI of the PEC facility over time

The ERoEI for a PEC facility is calculated similarly to the PV-E facility as the total hydrogen energy output divided by the total energy input cost. A full derivation of the calculations for the ERoEI of a PEC facility may be found in Section S2 (ESI†) and the full description of parameter choices may be found in Section S3 of the ESI.† The annual energy output is the thermal energy contained in the hydrogen produced from the PEC water splitting facility per meter squared of light collection area. It is the product of the PEC conversion efficiency, *μ*_PEC_, the PEC performance ratio, PR_PEC_, and the annual solar insolation, *S*, in kW h (m^−2^ of PEC active area) year^−1^. The performance ratio for PEC devices includes losses due to shading of the panel from dust and debris, and losses from temperature fluctuations.^27,65^ There is, however, no need to consider losses due to DC to AC conversion or generation and utilisation mismatch so the performance ratio for the PEC device is expected to be higher than for PV and AE modules. The energy input costs consist of the thermal embodied energy cost including the energy for manufacturing and decommissioning the facility by landfilling, just as for the PV-E system. The ongoing, operating energy cost of the PEC facility is the cumulative thermal energy cost of running the facility including gas handling and compression, module heating, and water supply management.

### 2.2. Input parameters values for the PV-E and PEC facilities

All input parameters except solar insolation are technological parameters that vary between facilities. These parameters in general may be expected to improve over time as technology and manufacturing techniques advance, but not over the lifetime for an existing facility. Average solar insolation (*S*), in contrast, depends on where a facility is located. Long-term average insolation varies from 700–900 kW h m^−2^ year^−1^ in high latitudes to 2500–2900 kW h m^−2^ year^−1^ at lower latitudes and at high elevations, considering factors such as abundance of clouds, atmospheric aerosol and moisture concentration.^66^ The average solar insolation used in this study will be an intermediate value of 1700 kW h m^−2^ year^−1^ consistent with much of the United States, southern Europe, South America, and southern Asia. 1700 kW h m^−2^ year^−1^ is also frequently used as a moderate irradiation in photovoltaic literature.^29,67,68^ The latest (4th) edition of the methodology guidelines on life cycle assessment of photovoltaic electricity only prescribes the assumption of optimal panel orientation, rather than any specific value for irradiation to use for analysis of systems based on average technologies, noting that the irradiation depends on location.^65^ A related factor is the effect of temperature on the modules, but in this work, this effect is assumed to be embedded in the average performance ratio and efficiencies of each technology.

#### Technological parameters for the PV-E facility simulation

The technological parameters for the PV-E facility simulation are for silicon-based photovoltaic and alkaline electrolyser facilities and summarised in Table 1. Base-case values are extracted from industry or internationally recommended standards^61^ and represent average current facility performance values. Favourable-case values are representative of the best presently published large-area devices with metrics appreciated to the present year 2023 where practical. Optimistic future-case parameters are compiled from literature estimates of future metrics or by applying a learning curve to devices over a 20–25 year period. This timeframe is commensurate with common facility lifetimes and with international reports that estimate scenarios up to the year 2050. Below, the values used or calculated and their sources for each input parameter are briefly summarized. A full description of the parameter choices and sources may be found in the ESI† Section S3.

**Table 1:** PV-E facility technological parameters

	Base case	Favourable case	Optimistic future case
PV facility parameter [unit]			
Silicon photovoltaic cells			
PV conversion efficiency (CE) [%]	21^b^^ ^^63^	26.8^71^	29^a^^ ^^73–75^
PV efficiency degradation rate [% of CE year^−1^]	0.7^65^	0.5^65^	0.3^76,77^
PV performance ratio [%]	80^65,84^	90^65,84^	95^a^^ ^^62^
PV upfront energy cost [kW h m^−2^]	788^a^^ ^^20,63,67^	537^b^^ ^^20,30,79^	322^b^
PV maintenance energy cost [kW h m^−2^ year^−1^]	7.9^a^^ ^^63^	5.4^a^^ ^^63^	3.2^a^^ ^^63^

AE facility parameter [unit]			
Alkaline electrolyser with nickel-based electrodes			
AE conversion efficiency [%]	65^81,82^	68^81^	76^47^
AE efficiency degradation [% of CE year^−1^]	1.50^57^	1.00^57^	0.25^57^
AE upfront energy cost [kW h m^−2^ of PV]	134^28^	119^b^^ ^^47,83^	49^b^^ ^^47,83^
AE operating energy cost [kW h m^−2^ year^−1^ of PV]	19^a^^ ^^3,47^	28^a^^ ^^3,47^	36^a^^ ^^3,47^

PV-AE effective combined parameters [unit]			
Overall conversion efficiency [%]	10.9^a^	16.4^a^	20.9^a^
Efficiency degradation [% of CE year^−1^]	2.0^a^	1.5^a^	0.37^a^
Upfront energy cost [kW h m^−2^ of PV]	922^a^	656^a^	371^a^
Operating energy cost [kW h m^−2^ year^−1^ of PV]	26.9^a^	33.4^a^	39.2^a^

a Values chosen by the authors as an average or calculation from multiple literature sources.

b Values taken from literature and appreciated to the present or future.

The average efficiency of newly installed PV panels is projected to reach 21% in the present year 2023 and this value is used as the base-case parameter.^63^ In fact, commercial panels with advertised efficiency up to 22.8–23.0% are now available from two manufacturers.^69,70^ The favourable-case parameter used is the record 26.8% efficiency for a single-junction silicon-based solar cell with an area of 274 cm^2 ^^71^ tabulated in the 2023 Solar cell efficiency tables (version 62).^72^ The optimistic future-case value is taken as approximately 29% which approaches the theoretical limits calculated by several sources for single-junction cells,^73–75^ and may be conservative for future multi-junction solar cells.^72^ The base-case PV efficiency degradation rate used comes from the 2023 (4th) edition of the methodology guidelines on life cycle assessment of photovoltaic electricity^65^ which recommends a degradation rate of 0.7% for mature module technologies. Furthermore, a degradation rate of 0.5% is recommended for use in sensitivity analyses,^65^ which is used here in the favourable case. For the optimistic-case parameter, a value of 0.3% is taken from the approximate median degradation rates among recent silicon-based devices in a pair of meta-analyses on PV degradation.^76,77^ The base-case and favourable-case PV performance ratios are reported to lie between 80–90%.^65^ Although a direct PV-coupled electrolysis facility may not require DC–AC conversion, a PV module under optimal conditions may still need a DC–DC converter to ensure the optimal voltage is supplied; this tends to result in a 5–10% power loss,^62^ so 95% is chosen as the optimistic-case bound.

Few sources report the embodied energy cost for constructing PV facilities. 750 kW h m^−2^ is used in this work as an approximate value for the construction energy, given the limited availability of sources, including from de Wild-Scholten in 2013 (739 kW h m^−2^),^67^ Goerig & Breyer of (750 kW h m^−2^) between 1974–2010,^20^ and Raugei *et al.* (767 kW h m^−2^) in 2017.^63^ We use here for decommissioning the approximately 5% energy costs reported for landfilling CdTe thin film modules without recycling.^78^ yielding a final upfront embodied energy cost in the base case of 788 kW h m^−2^. An embodied energy of 537 kW h m^−2^ is used as the favourable-case present parameter calculated by applying a learning rate of 12%^30^ for three doublings of cumulative installed capacity.^79^ The optimistic future-case value is calculated as 322 kW h m^−2^ assuming a further four doublings of cumulative installed capacity in the next 20 years. We conservatively chose a PV maintenance energy cost of 1% of the PV embodied energy costs in each parameters case following the 2017 analysis by Raugei *et al.*^63^ Primary literature is, however, difficult to source and other reports on technical risks note clearly that PV inverter failure rates are rarely disclosed by manufacturers.^80^ In the base case, the maintenance energy cost is 7.9 kW h m^−2^ and the favourable and optimistic future-case values are 5.4 kW h m^−2^ and 3.2 kW h m^−2^ respectively.

The base-case and favourable-case AE conversion efficiency values are taken as 65% from a 2018 IRENA report for alkaline electrolysers that was predicted to rise to 68% in 2025.^81^ This progress agrees with predictions in an expert elicitation study from 2017 that stated system efficiencies would reach 60 to 65% in 2020.^82^ A separate 2020 IRENA report uses 76% for future conditions in 2050 which is taken as the optimistic-case value.^47^ There are few specific reports of electrolyser efficiency degradation rate. Degradation of electrolyser efficiency between 0.10 to 1.50% was reported in one 2015 study^57^ for eleven commercial alkaline electrolysers. Reviews on alkaline water electrolysers published in 2018,^58^ 2019,^48^ and 2021^59^ have since cited these values. The base-case value here is chosen as 1.50% annual degradation whereas the favourable-case is 1.00% degradation and 0.25% annual degradation is used as the optimistic-case value.

For the AE embodied energy cost in construction, energy intensities of 2.69 × 10^6^ MJ MW^−1^,^28^ and 2.79 × 10^6^ MJ MW^−1 ^^22^ have been reported. For the base-case parameters, after factoring a 21% conversion efficiency and 0.80 performance ratio, an AE with embodied energy including decommissioning of 134 kW h m^−2^ of PV is needed. Considering a 12% learning rate,^83^ and a 2020 IRENA report showing that water electrolysis doubled in capacity approximately four times between 2015 and 2023 and may double in capacity another eight times in the next 20 years,^47^ the base-case energy intensity of 2.79 × 10^6^ MJ MW^−1^ is reduced to 1.67 × 10^6^ MJ MW^−1^ and 0.60 × 10^6^ MJ MW^−1^ in the favourable and optimistic future cases respectively. Therefore, in the favourable case, the embodied energy would be 119 kW h m^−2^ of PV and in the optimistic case, the final embodied energy of the AE would be 49 kW h m^−2^ of PV. The operating energy cost of the AE is taken here as the adiabatic compression of hydrogen to 200 Bar of approximately 10% of the energy stored in the hydrogen, yielding an energy cost of 19 kW h m^−2^ year^−1^ for the base case. Future improvements in compression energy costs will likely be physically limited. Therefore, using the average 10% cost of the hydrogen energy produced for the favourable and optimistic-case systems leads to compression energy costs of 28 and 36 kW h m^−2^ year^−1^ respectively. These values are larger because more hydrogen is produced.

#### Technological parameters for the PEC model

Similar to the PV-E facility, most input parameters to the PEC model are technological parameters that could vary in practice between facilities. Typical ranges of values for the technological parameters for a PEC facility obtained from literature are summarised in Table 2. Few studies have been conducted with large-scale PEC devices so the parameters chosen are inherently more uncertain. Compared to Si photovoltaic cell technology, for instance, the ultimate materials composition of future PEC water splitting devices is also uncertain and may affect the energy parameters. In these models, the base-case parameters reflect devices consisting of only earth-abundant materials. The favourable-case parameters reflect higher-performance devices that use precious metal co-catalysts, while the optimistic future-case parameters are for optimised performances proposed in the literature for facilities 20–25 years in the future where available. This timeframe is chosen as most likely to encompass significant technological progress, but a discussion of this progress in reference to global climate change mitigation goals is beyond the scope of this work. Below, the values used or calculated and their sources for each PEC input parameter are briefly summarized. A full description of the parameter choices and sources are in the ESI† Section S3.

**Table 2:** Technological parameter ranges for a PEC facility

Input parameter [unit]	Base case (Earth-abundant materials)	Favourable present case (precious co-catalysts)	Optimistic future-case parameters
PEC conversion efficiency [%]	3^89^	5^89^	10^a^
PEC efficiency degradation rate [% of CE year^−1^]	10^77,94^	4^23^	2^23^
PEC performance ratio [PR]	0.85^27,61,97,98^	0.90^23^	0.95^27,61,97,98^
PEC upfront embodied energy [kW h m^−2^ of active area]	347^23^	516^23^	431^a^
PEC maintenance energy [kW h m^−2^ of active area]	33^a^	49^23^	41^a^

a Values chosen by the authors as an average or calculation based on literature sources.

There are few reports of the conversion efficiency of large-scale PEC water splitting devices in the literature.^42,85–88^ The Artiphyction project, completed in 2015, yielded the first large-scale 1.6 m^2^ PEC prototype using CoPi-catalysed molybdenum-doped BiVO_4_ which showed initial conversion efficiency of 3% and concluded that further engineering efforts were needed to improve fluid dynamics and to discover better photo-electroactive materials.^89^ This value of 3% conversion efficiency will be chosen as the base-case parameter. 5% conversion efficiency was their programme target and will be chosen as the favourable-case parameter. Further examples of PEC devices on large-scale demonstration can be found in relevant reviews,^11,13^ and at the Solar Fuels Database compiled by EPFL.^45^ Predicting near-future conversion efficiency is, however, highly challenging because few PEC devices are in operation and the future materials and configurations of devices may vary greatly from present prototypes. 10% is taken as an illustrative, optimistic future-case conversion efficiency for large-scale PEC devices in the next 20 years, corresponding to, for reference, five doublings of capacity and a 15% learning curve. This value is also justified by the demonstration of PEC devices at lab scale with ∼8% conversion efficiency.^90,91^

Most studies of experimental PEC devices in the literature only show or test for PEC photoelectrode stability over 1 day or less,^92,93^ after which time there is already significant degradation. One demonstration of a photoelectrochemical cell was a 50 cm^2^ hematite photoanode in tandem with two silicon heterojunction solar cells that reported a very stable performance of 0.04% annualised degradation over 42 days.^94^ Upon close inspection, however, there is a drop when considering early plateau regions and performance later on which indicate a 10% drop in conversion efficiency over the same time. This value will be chosen as the base-case parameter. Other examples of particulate BiVO_4_ and photoanodes tested in vanadium-saturated electrolyte showed 1000 and 500 hours of stability respectively.^95,96^ For the theoretical facilities simulated by Sathre *et al.*,^23^ the worst case lifetime of the system is 5 years which corresponds to a 4% annual linear degradation rate for a facility that reaches the end-of-life when efficiency is reduced by 20% from the initial value. Their base-case lifetime was 10 years, corresponding to a 2% annual linear degradation rate and these values are chosen here as the favourable-case and optimistic future-case parameters respectively. These metrics are illustrative estimates of the degradation rates of PEC prototypes in the future. The performance ratio for PEC devices includes losses due to shading of the panel from dust and debris, and losses from temperature fluctuations.^27^ There is, however, no need to consider losses due to DC to AC conversion or generation and utilisation mismatch so the performance ratio for a PEC module could be expected to reach approximately 0.85–0.95.^27,61,97,98^ 85% will be used as the base-case value and 95% as the optimistic-case value. We chose 90% as an average favourable-case performance ratio, which agrees with a calculated estimate of the performance ratio from the expected energy output.^23^

For a base-case performance facility using only earth-abundant photoabsorbers and catalysts, Sathre *et al.* estimated the total embodied energy to cost 347 kW h m^−2^, whereas their moderate energy intensity facility with some precious metal catalysts costed 516 kW h m^−2 ^^23^ and is chosen as the favourable-case performance parameters for this model. Because these parameters are already predictions, no learning curve is applied although the work was published in 2016. For the optimistic-case future embodied energy cost, the facility is assumed to improve over 20 years to an average of the previous cases of 431 kW h m^−2^. It is yet to be determined whether low-cost earth abundant PEC catalysts with improved conversion efficiency or higher-cost precious metal catalysts with lowered costs may ultimately be more effective on an overall net-energy basis in the future. The energy for handling and compressing the gas, the energy for module heating, and for managing water supply was reported by Sathre *et al.* to total an energy cost of 49 kW h m^−2^ year^−1^ for the favourable-case performance system used here.^23^ This value is comparable with but larger than the 39.2 kW h m^−2^ year^−1^ effective maintenance cost for the PV-AE system. Proportionally, the base-case and optimistic future-performance case maintenance energy costs are 33 and 41 kW h m^−2^ year^−1^ respectively.

## 3. Results & discussion

### 3.1. Energy balance of the PV-E facility

#### PV and PV-E facility scenarios

**Fig. 2:**
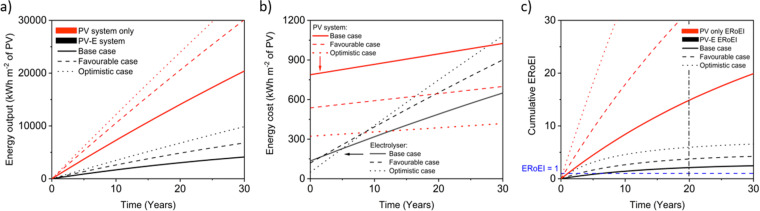
Energy metrics over time for simulated PV facilities (red traces) and PV-coupled electrolyser facilities (black traces) for (a) output energy production, (b) energy input costs, and (c) ERoEI for electricity and hydrogen production. The shaded envelopes show the range in the favourable to unfavourable parameters about the base-case simulation.

We first validate our model on an isolated PV system for electricity output. Energy output is shown in Fig. 2a (converting electrical energy to thermal through dividing by 0.38, as in Raugei *et al.*^63^), the cumulative thermal energy input in Fig. 2b, and ERoEI in Fig. 2c. The electrical output ERoEI of a PV system after 20 years is found to be 14.9 and the EPBT (when the ERoEI crosses 1) to be 1.1 years in the base case (red traces). This base-case EPBT estimate is within the range of times reported in the most up-to-date Fraunhofer Photovoltaics Report from September 2022 using ratios harmonised by considering a 0.35 grid efficiency for converting PV yields.^84^ That study reports the present EPBT as 1.1 years in northern Europe to 0.9 years in southern Europe and as low as around 0.5 for near the equator for 19.9% efficient silicon PV rooftop systems made in China.^84^ The ERoEI of 14.9 is also consistent with the conservative ERoEI range of ∼19 for PV electricity harmonised with a ratio of 0.3 summarized by Murphy *et al.* in their review that addressed consistent energy units for the ERoEI of fossil-fuel and renewable energy sources.^64^

Next, we calculate the ERoEI and EPBT of a coupled PV-E facility. The cumulative output energy of the PV-E facility is shown in Fig. 2a. The cumulative energy output in each case increases over time, but at a decreasing rate, due to the conversion efficiency degradation. Fig. 2b shows the cumulative energy input costs for the separate PV and AE components in each case. The electrolyser size and therefore its embodied and operating energy costs are dependent on the PV electricity output. When the favourable performance costs are simulated, the large PV output energy requires more electrolyser capacity. When the unfavourable input costs are simulated, the small PV output energy requires less electrolyser operation energy costs. The ERoEI over time is shown in Fig. 2c for the hydrogen produced by the PV-E facility. The base-case hydrogen output ERoEI for the full PV-E system after 20 years is 2.1 and the EPBT is in 6.2 years. As Fig. 2b shows, the energy input costs for the PV system are dominated by the one-time upfront embodied energy costs, but the operating energy costs play a greater role for the electrolyser component.

#### Parameters sensitivity analysis of the PV-E system

Sensitivity analysis of the input parameters of the PV-E model is necessary because of the high number of input parameters in our analysis (nine in total). It is important to consider the relative effect of changing each parameter to identify priorities for improvements. Here, we analyse sensitivity in two ways, first by varying one parameter with others kept at the base-case or favourable values, and then by performing high-throughput Monte Carlo analyses randomising multiple parameters.

**Fig. 3:**
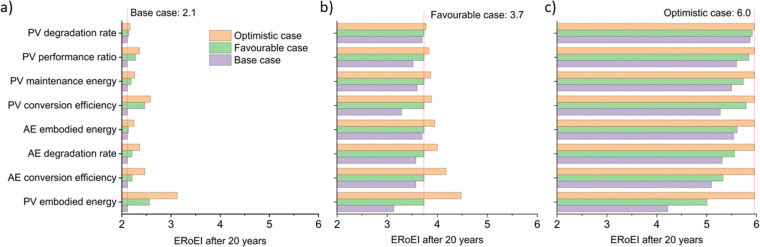
Parameters sensitivity analysis of the ERoEI after 20 years for the PV-E facility for (a) varying the individual parameters to their favourable and optimistic cases while keeping the remaining parameter values at their base case estimates, (b) varying individual parameters to the base and optimistic cases while keeping the remaining parameter values at their favourable case estimates, (c) varying individual parameters to the base and favourable cases while keeping the remaining parameter values at their optimistic-case estimates.

Fig. 3a shows the calculated ERoEI of a base-case PV-E facility after 20 years when each input parameter is varied individually to its favourable or optimistic future-case values. Reducing the PV embodied energy is the change that increases ERoEI the most (2.6 and 3.1 respectively after 20 years, up from 2.1 in the base case). Similarly Fig. 3b shows the effect of varying parameters to the base and optimistic cases for a favourable-case facility and Fig. 3c uses the optimistic-case facility as the reference scenario.

The ERoEI after 20 years is 3.7 and 6.0 with an EPBT of 2.7 and 1.2 years when all input parameters are set at their favourable and optimistic values. The ERoEI has an effectively linear dependency on all of the parameters, with a minor non-linear change in ERoEI with changing PV embodied energy cost (shown in Fig. S3 (ESI†) for ERoEI deviations from the favourable and optimistic cases). PV embodied energy is the parameter with the greatest effect on PV-E system net-energy balance.

**Fig. 4:**
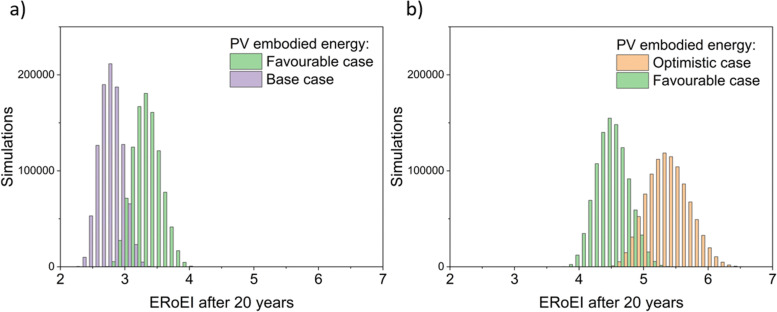
Histograms of ERoEI after 20 years setting the PV embodied energy at the indicated bounds and selecting all other parameters at random within their specified ranges (defined in Table 1) for (a) between the base and favourable cases, and (b) between the favourable and optimistic cases; 1000 000 simulations for each profile.

Fig. 4 presents the results of a Monte Carlo simulation to show the typical ERoEI when setting the PV upfront energy and allowing the values of other parameters to randomly sample values within their specified ranges. The result of one million simulations (chosen as a manageable computational value) for each set of parameters are plotted as histograms. In Fig. 4a, a favourable PV upfront energy improves the ERoEI by approximately 1 over the base-case value. In Fig. 4b, the range in possible ERoEI values after 20 years agrees with one literature estimate (for a 30-year lifetime PV plant and 10-year lifetime AE)^22^ for future PV-E facilities to reach an ERoEI of 4 (also matching the peak of scenarios for a Monte Carlo simulation varying all parameters between their base and optimistic cases shown in Fig. S4, ESI†). In all cases, the PV-E simulations show ERoEI greater than unity but a favourable PV upfront energy cost is necessary for the ERoEI to be greater than 3.

### 3.2. Energy balance of the PEC water splitting facility

**Fig. 5:**
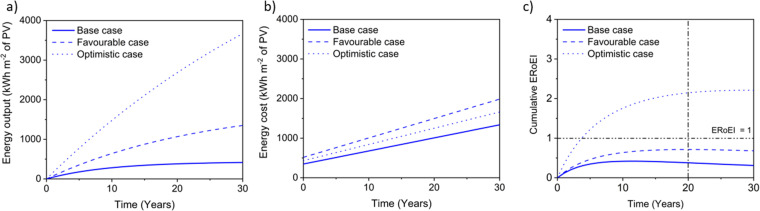
(a) Cumulative PEC output energy, (b) input energy costs, and (c) ERoEI for hydrogen output for the optimistic, favourable, and base-case input parameters.

Fig. 5a shows the cumulative PEC hydrogen output energy and Fig. 5b shows the cumulative PEC input energy for the different cases defined in Table 2. The energy input cost is always larger than the output energy gained for the base and favourable cases. Only in the optimistic case does the energy output surpass the energy input costs. Fig. 5c shows the ERoEI over time for all three cases. The peak in ERoEI is 0.42 for the base case and occurs after 11 years. In the favourable case, the ERoEI peaks at 0.71 after 20 years. These results are small because of the technical parameters of such an early-stage technology. They are also in line with the work of Nishiyama & Domen who reported a 100 m^2^ pilot plant based on photocatalytic sheet technology,^99^ which over a 3-month period had a STH efficiency varying from 0.5% to 0.2% and an ERoEI of 0.82 when considering only the energy output and energy required to run gas pumps (without upfront construction or decommissioning energy costs). Only when considering optimistic limits for its parameters will the PEC facility have a more competitive ERoEI after 10 years of 1.8 (2.1 after 20 years). This case matches the performance of the present base-case for PV-E facilities, although in the time it takes for PEC technology parameters to reach their optimistic values, PV-E technology will have improved as well according to some learning curve.^20,100^

#### Sensitivity analysis for ERoEI over time of a PEC system

**Fig. 6:**
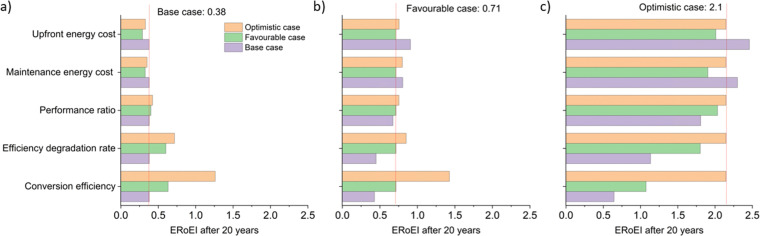
Sensitivity analysis for parameters affecting the PEC facility starting at (a) the base-case scenario (ERoEI of 0.38), (b) the favourable-case scenario (ERoEI of 0.71), and (c) the optimistic-case scenario (ERoEI of 2.1).

Fig. 6 shows a sensitivity analysis varying each parameter of the PEC system. Fig. 6a shows the variation in the base-case ERoEI after 20 years when the input parameter values are increased individually to their favourable and optimistic values. The EPBT in all cases when most parameter values are held at their base cases are not available because the ERoEI never surpasses unity. The parameters with greatest impact are the conversion efficiency and the degradation rate in all cases, including for varying the scenarios from the favourable case (Fig. 6b), and from the optimistic case (Fig. 6c) indicating they are the most critical to improve to ensure higher ERoEI. The optimistic-case peak ERoEI was calculated to be 2.2 after 29 years (2.1 at 20 years and 1.8 at 10 years) with an EPBT of 3.7 years. The effect of changing each parameter is mostly linear (shown in Fig. S5 (ESI†) for ERoEI deviations from the favourable and optimistic cases).

One often cited advantage of PEC facilities is their potential for lower capital costs due to their integrated nature compared to PV-E systems. In these models, the embodied energy in the base and favourable cases is lower for the PEC compared to the effective total embodied energy for the PV-E system (Table 1). The embodied energy cost of the optimistic future case facilities is, however, smaller for the PV-E system because of its learning curve improvements as a function of projected increases in installed capacity. The PEC facility may lose its advantage of being a simpler construction if there continues to be a lack of large-scale experimental PEC studies and developments. We note in any case, that previously reported PEC embodied energy^23^ is comparable in magnitude to that of PV-E facilities^20,67^ and we calculate that embodied energy is not amongst the key factors limiting the overall ERoEI of PEC facilities. We speculate that for PEC facilities, the increased need for liquid management offsets the simplicity of electrical connections and physical integration compared to PV-E (which may not be a large expense because of the maturity of PV-E).

**Fig. 7:**
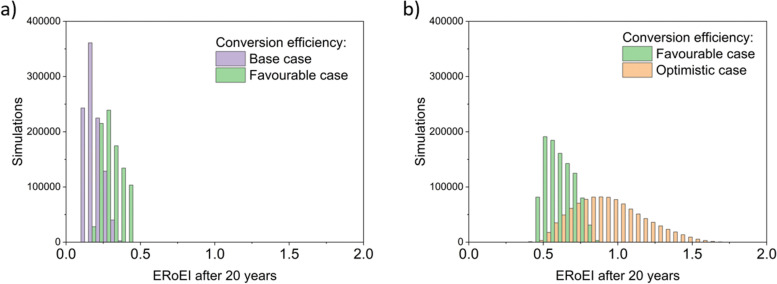
Histograms of the ERoEI after 20 years for a modelled PEC facility with conversion efficiency (CE) indicated; 1000 000 simulations each profile, (a) randomised parameters between the base case and favourable case, (b) randomised parameters between the favourable case and optimistic case.

The histograms of Monte Carlo simulations in Fig. 7 illustrate the probable outcomes when the values of PEC conversion efficiency are controlled and the other parameter values are randomized within their ranges between the base and favourable cases for Fig. 7a and between the favourable and optimistic cases for Fig. 7b. The most common ERoEI after 20 years is ∼0.4 when all parameters are varied randomly between their base and optimistic cases as shown in Fig. S4 (ESI†). Only when conversion efficiency is set at its optimistic future value does the ERoEI of the water splitting facility after 20 years reach values around 1. These values are still significantly less than the ERoEI for SMR, 2.47,^52^ and are also lower than the previously published estimation of the ERoEI of theoretical PEC facilities of 1.74–2.34 depending on solar insolation.^23^ In that work, the base case active components also had a 10% STH efficiency and 10-year lifetimes but were continually replaced over a 40-year facility lifespan, with the re-use of the balance of systems.

### 3.3. The effect of recycling on the net-energy balance calculations

An understanding of the energy impact of recycling is important for evaluating future technologies. Using recycled material to make new components lowers the energy input costs of hydrogen production facilities by avoiding energy expenditure in extraction of primary materials. Recycling the materials results in an increase in energy cost for the recycling process of spent components, but past life-cycle analyses of renewable hydrogen production facilities often do not consider recycling because the low volumes of spent components generated to date^101^ has resulted in a lack of available data on recycling routes.^102^ If renewable hydrogen production facilities proliferate, spent modules will accumulate and recycling data should become increasingly available.

To include recycling in the simulations, the energy cost of constructing the modules from recycled material replaces the energy cost for constructing the materials from new material. The energy cost of recycling the materials also replaces the energy cost of disposing in landfill. This decrease in energy cost due to the replacement of new material with recycled material offsets the increase in energy cost for the recycling of the waste. For certain materials here, the lack of reported data will be bridged by using values for recycling of elements generically and not specific to water splitting technology. Only components of the modules that are currently commonly recoverable such as glass and metals will be considered here. We also assume that the modules produced from recycled material have the same initial performance efficiency as the non-recycled modules. In practice, the recycled modules may have a lower initial conversion efficiency depending on the extent of material purification or in fact higher initial conversion efficiency as incremental improvements to the technology are made in the time it takes for a module to complete one lifecycle and be replaced.

Apart from energy balance considerations, developing widely commercialised renewable hydrogen production is challenged by the need for avoiding materials scarcity in the near future.^103,104^ For instance, high-performance, but high-cost electrolyser designs use precious metals including platinum, iridium or ruthenium as catalysts.^23,105^ The most common PV module in use today, the crystalline silicon PV, uses silver for electronic contacts^105^ and the most common electrolyser deployed today, the alkaline electrolyte electrolyser, uses nickel for its electrodes.^102^ High-performance PEC modules could rely on scarce elements such as gallium, arsenic, cadmium, and tellurium for light absorption.^23^ The conductivity of the glass electrodes relies on transparent conducting oxides made from tin^106^ or indium,^107^ which is a similar materials constraint for flexible organic PV modules^108^ compared to silicon PV modules. Recycling is important for mitigating materials depletion, as has been explored for electronics waste,^109^ but the available data on recycling PV-E and PEC components is extremely limited.

#### Simulating recycled PV-E facilities

To simulate recycled PV-E facilities, metrics for the recyclability and energy costs to recycle each material in a typical module are needed. The additional energy costs to recycle, the energy savings from using recycled material, and the proportion of each component to the favourable-case energy cost were extracted from published reports and tabulated in Table S1 (ESI†). The values are representative and particular facilities may have differing values for proportions of materials. Critically, upon analysis of the energy costs to recycle and the energy savings when producing the recycled material in place of virgin material, recycling provides a net-energy benefit for every material investigated. Furthermore, although non-recoverable plastics make up a non-insignificant portion of the weight of facilities, these are often incinerated to generate electricity,^110,111^ and will be assumed here to be net-negligible.

In this work, the final recalculated embodied energy of the modules with recycling comes out to 316 kW h m^−2^ for the Si PV and 60 kW h m^−2^ of PV for the AE compared to 537 kW h m^−2^ for the Si PV and 119 kW h m^−2^ of PV for the AE in the non-recycled, favourable performance case. These values are reductions of 41% for the PV and 50% for the AE. Briefly, the materials for the AE were considered for the stack and balance of plant reported in a 2021 life-cycle assessment.^112^ The energy savings for AE is high because recycling nickel, copper, and steel saves significant amounts of energy (91%,^113^ 90%,^113,114^ and 81%^115^ respectively and tabulated in Table S1, ESI†). The materials inventory assumed here does have uncertainties and published life-cycle assessments of alkaline electrolysers are not able to disclose all their components exactly.^112^ The PV also has a high energy savings for recycling because the silicon energy cost is reduced by 40% and makes up a majority (95%) of the original energy cost of the modules. Glass makes up a large component (75%) of the PV module weight, but a small portion of the overall energy cost.

**Fig. 8:**
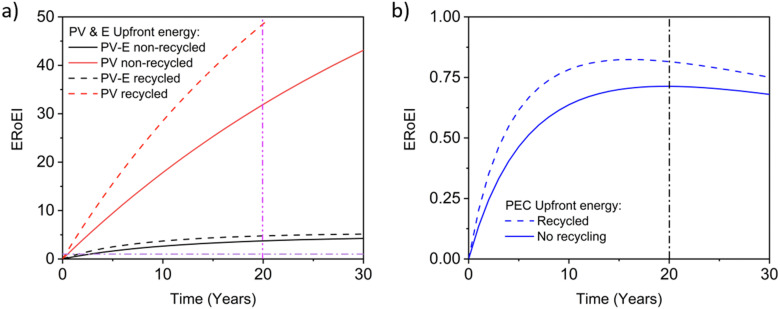
ERoEI over time using recycled modules compared to without recycling for (a) the favourable case PV and PV-E facility and (b) the favourable case PEC facility. (ERoEI = 1 and Time = 20 years indicated with dash dot lines.)

In Fig. 8a, the recalculated ERoEI over time for the favourable-case PV-E system considering recycling is compared to the simulation without recycling for both the PV system alone and for the full PV-E water splitting system. The electricity ERoEI for the PV after 20 years is now 48.6 (up from 31.9, a 52% increase) and EPBT for electricity still occurs well before 1 year. The hydrogen ERoEI for the PV-E after 20 years and EPBT, 4.8 and 1.6 years, are 30% and 41% better than the 3.7 ERoEI and 2.7-year EPBT for the system without recycling. This ERoEI improvement would firmly establish the ERoEI of the recycled PV-E system as significantly better in energy terms than conventional SMR.

#### Simulating recycled PEC facilities

For PEC facilities, the favourable present case embodied energy cost is 516 kW h m^−2^.^23^ Table S2 (ESI†) considers recycling for the glass, aluminum, and light absorbing photocatalysts. The energy costs of recycling metal oxides is not readily available, so the effect of recycling PEC photocatalysts is estimated here by considering the light absorbers in CdTe photovoltaics. Both materials are thin films that require separation from glass, chemical leaching into solution, and then isolation by, for example, electrochemical means.^116,117^ The recycling of CdTe thin films has been thoroughly studied due to the high value of tellurium and high toxicity of cadmium, leading to the European Commission to regulate their waste treatment.^118^ Under extensive recycling, an average of 23% net energy savings was reported (∼270 MJ m^−2^ in savings compared to 1190 MJ m^−2^ for CdTe PV modules with no recycling).^78^ This proportionality is applied to the light absorbers in PEC systems to estimate the new energy intensity of the photocatalyst component. The final recalculated embodied energy of the entire PEC system with recycling is 329 kW h m^−2^, a savings of 36%. The recalculated favourable case ERoEI of a PEC facility including recycling is shown in Fig. 8b compared to the ERoEI without recycling. Recycling does increase the ERoEI after 20 years by 14% from 0.71 to 0.82. This improvement is proportionally less than the underlying recycling energy savings rate due to the large PEC maintenance energy.

In both simulations of recycled PV-E and PEC facilities, the materials recovered after decommissioning are assumed to be used in subsequent facilities and not reincorporated into the original facility. Reincorporation would increase the energy output due to a sustained conversion efficiency, but is offset by increased ongoing energy inputs over time.

### 3.4. Discussion

The hydrogen ERoEI for all the PV-E and PEC system scenarios modelled here are compared in Table 3. For comparison, one literature report calculated the ERoEI of SMR to be 2.47 and EPBT to be 8.1 years,^52^ but very few studies on SMR report energy balance, including those that perform a technoeconomic analysis^119^ or life-cycle assessment.^120^

**Table 3:** Summary of energy balance simulation results for our modelled PV-E and PEC facilities. A reported value of the ERoEI for SMR is shown for comparison. NA – not available

Hydrogen production technology ERoEI (EPBT)	Base case	Favourable case	Optimistic case
PV-E ERoEI after 20 years	2.1 (6.2 years)	3.7 (2.7 years)	6.0 (1.2 years)
With recycling	2.9 (3.5 years)	4.8 (1.6 years)	6.9 (0.7 years)
PV-E ERoEI after 10 years	1.4 (6.2 years)	2.7 (2.7 years)	4.6 (1.2 years)
With recycling	2.1 (3.5 years)	3.7 (1.6 years)	5.8 (0.7 years)
PEC ERoEI after 20 years	0.38 (NA)	0.71 (NA)	2.15 (3.7 years)
With recycling	0.43 (NA)	0.82 (NA)	2.45 (2.4 years)
PEC ERoEI after 10 years	0.42 (NA)	0.64 (NA)	1.76 (3.7 years)
With recycling	0.51 (NA)	0.78 (NA)	2.16 (2.4 years)
SMR ERoEI (literature)	2.47^52^ (8.09 years)		

Recycling improves the ERoEI for the PV-E facility in most cases between 30–40%, but only approximately 10–20% for the PEC facility. Embodied energy values for recycled base-case and optimistic-case facilities are chosen with the same proportions as the energy changes calculated for the favourable-case facilities (Tables S1 and S2, ESI†).

#### Key improvements for PV-E water splitting

PV-E water splitting already shows an energy balance that is comparable to SMR for its base case without recycling. Trends in continuously improving manufacturing efficiency are likely to push down the upfront energy cost, as volumes of device manufacturing grows.^47^ Therefore PV-E energy balance should improve further in efficiency and in annual volumes of hydrogen generated.^100^ The rates of cost reduction would also increase for faster rates of societal energy transition to clean energy technology.^100^ A combination of learning curve improvements to the energy output and input values along with increased recycling will likely improve the energy-balance of PV-E water splitting in the near future.

#### Key improvements for direct PEC water splitting

PEC water splitting, meanwhile, is further from viability in energy terms. Apart from recycling, a focus on parameters that have the greatest energy balance impact will be essential. Favourable-case parameters are not sufficient for the facility to have an ERoEI greater than 1. If conversion efficiency is taken to its optimistic limit, the ERoEI does rise to 1.4. To surpass an ERoEI of 2, the conversion efficiency and degradation rates must be near the optimistic bounds whereas the other parameters are less critical. To become competitive with SMR and PV-E water electrolysis, parameter values more favourable than those simulated in the optimistic case are needed.

Considering the roughly 70-year history of photovoltaics may be informative in predicting the future of PEC water splitting. In 1954, a photocell of crystalline Si PV was first demonstrated with a laboratory efficiency of 6% and 0.5% commercial efficiency.^121^ Laboratory efficiencies then surpassed 20% in 1985 and commercial PV installations experienced exponential growth from the 1990s.^122^ By 2010, global commercial PV capacity was 40 336 MW and growing annually by an average of 34% to over 19 times that at 773 200 MW in 2020.^123^ PV-coupled electrolysis was noted to be a developing application in a 2010 review of alkaline water electrolysis by Zeng and Zhang, which also outlines succinctly the 100-year history of commercial water electrolysis starting with large-scale plants for producing ammonia.^124^

Comparatively, the development of direct solar-to-hydrogen PEC water splitting has been delayed. It was first demonstrated in 1972 using TiO_2_,^125^ but was limited to a theoretical maximum conversion efficiency of 2.2% due to its large bandgap. The record performance for a TiO_2_-based device is only 1.1% STH efficiency and 2.6 mA cm^−2^ under 1 sun illumination.^126^ Since then, III–V semiconductors at laboratory-scale have reached 19% efficiency in 2018 for monolithic device architectures with precious metal co-catalysts that drive hydrogen and oxygen evolution simultaneously.^15^ In 2023, a demonstration of a 4 cm × 4 cm III–V semiconductor-based water splitting device showed an average 9.2% STH conversion efficiency under concentrated illumination and self-heating to ∼70 °C; this device showed a promising 6.2% STH conversion efficiency in an outdoor test under a natural solar light capacity.^127^ For lower-cost metal oxide semiconductors, the state-of-the-art efficiency for PEC devices remains at only about 3.0% in 2018.^128^ Tandem PEC-PV devices such as BiVO_4_-based photoanodes coupled to perovskite or III–V semiconductor PVs are more promising and are showing efficiencies from 6.2% with iron-nickel cocatalysts^129^ to 8.1% with cobalt phosphate cocatalysts.^130^ Cobalt phosphate in particular is a well-studied, relatively earth-abundant material that can be nanostructured into highly effective and 90% optically transmissive films.^131^ Along with improving cocatalysts, developing stronger light absorbing materials may be a key route to viable devices. Si-based and III–V semiconductor based photocatalysts embedded in protection layers have shown high efficiency but may suffer from short operating lifetimes and high cost.^132^ As examples, recent publications investigating the stability of GaAs in acidic or alkaline electrolyte^133^ and the energetic limits to 2.0 eV bandgap CuInGaS_2_ (CIGS) absorbers that could be embedded in PEC devices^134^ may help to guide research in improving earth-abundant light absorbing materials. If high-efficiency PEC devices are developed, potentially with small amounts of precious metal co-catalysts, the theoretical efficiency limits for PEC devices may be achieved which has been calculated to exceed optimised PV-coupled electrolysis.^135^

Apart from efficiency, the degradation rate of the PEC system is the other critical limiting factor for positive energy balance over its lifetime. Many researchers focus on using metal oxide-based technologies for PEC over traditional semiconductors such as those used for PV (crystalline Si and III–V semiconductors) because metal oxides are more stable in aqueous electrolyte, and additionally, of lower cost to produce.^13^ Regardless, stability studies for PEC devices have seldom extended more than 1–2 months so even the estimate of a 5-year lifetime with a 4% annual degradation rate^23^ is optimistic. A 2022 solar fuels roadmap suggests that a lifetime greater than 10 years and solar-to-hydrogen efficiency near the theoretical limit of 22% may be needed for commercial viability.^10^ Even so, the design principles that have aided silicon PV advancements are likely to promote more rapid development of PEC technology such as attention to minimizing recombination by control of microstructure. As Jacobssen^16^ suggests, however, unless PEC water splitting can be developed to operate near its limits, water splitting as an application may be better addressed by PV-coupled electrolysis. In the time needed for PEC technology to catch-up, PV-E will have further improved and potentially established itself as the default commercial route to hydrogen production by direct solar conversion.

#### Limitations to our simulations

Electricity grid integration and on-site battery storage for improved operability are possible elements of a practical solar-driven hydrogen production facility. The net-energy balance of this work could be improved by appropriate systems engineering, but the simplicity of our energy balance model (Fig. S1, ESI†) is an important starting point. A standalone facility is contained and so the energy costs are more simply quantified. Considering grid connection leads to variability depending on local electricity mix and price. There are also similar configurations of PV-E and PEC devices that we did not explore such as hybrid and solar-concentrated systems that were beyond the scope of this work. This work focuses on net-energy balance, although monetary cost competitiveness is also crucial for commercial adoption of any technology. Cost analyses available in the literature include a recent work comparing PV-assisted solar hydrogen generation which showed that direct solar hydrogen generation could be cost competitive with PV-coupled electrolysis in limited scenarios.^136^

Apart from the limitations in scope, this work was mainly limited by the data available and we acknowledge that the conclusions may be influenced by those. In general, the limited data for certain inputs may lead to a smaller than realistic range between the unfavourable and favourable cases of parameters. Metrics from large-scale PEC facilities are limited to theoretical studies, with experimentally verified parameter values only arising from laboratory-scale devices, leading to uncertainty in the inputs. Recycling data is available for all the elements studied here, although the application and waste material being recycled may be for entirely different systems than PV-E and PEC modules. Materials recycled in municipal waste streams are more likely to be mechanically crushed and separated instead of manually separated leading to lower recycling recovery rates and higher energy expenditures.^109^

## 4. Conclusions

We have shown that a system-level consideration of the energy balance of hydrogen production facilities is useful to compare technologies and determine critical parameters, and may be used to guide research development. In the base-case simulation here, the energy return on energy invested (ERoEI) of photovoltaic-coupled electrolysis (PV-E) is 2.1 after 20 years of operation and 3.7 in the favourable-case simulation, exceeding that of hydrogen produced using steam methane reforming. In contrast, photoelectrochemical (PEC) hydrogen production consumes more energy than is produced leading to an ERoEI less than 1, even in the favourable present-case facility due to low initial and fast degradation of its solar conversion efficiency. Monte Carlo simulations using published ranges of parameters demonstrated that for PV-E facilities, the upfront energy cost of the PV was the most impactful parameter, while for PEC facilities, conversion efficiency and degradation rate are the most critical. PV-E facilities reach an ERoEI after 20 years of 6.0 in the optimistic future case, while PEC water splitting facilities could show an ERoEI after 20 years of 2.2 and an energy payback time of 3.7 years in an optimistic future scenario. Projecting when PEC technology will have progressed to that point is, however, a significant challenge due to its low technology readiness level and is a major drawback of PEC water splitting compared to PV-E.

Green hydrogen production facilities are needed as soon as possible to reduce the carbon intensity of industry, as well as to enable hydrogen to become an energy vector in a low carbon emissions energy system of the future. These new facilities should use materials efficiently and be designed for recycling or reuse at the end of their operational lifetime. Recycling could play a vital role in the sustainability of this new infrastructure by reducing the upfront energy costs between 30 to 50% and increasing the ERoEI by 10 to 40%. We suggest that research in green hydrogen technologies with a greater emphasis on positive energy balance would help to accelerate the development of green hydrogen production and help decarbonise the future energy system.

## Data availability statement

The data presented in this study are available and may be obtained by contacting the corresponding authors.

## Conflicts of interest

There are no conflicts of interest to declare.

## Supplementary Material

EE-017-D3EE02814C-s001
