# Supplementary material for: Comparing the net-energy balance of standalone photovoltaic-coupled electrolysis and photoelectrochemical hydrogen production
Source: Energy Environ Sci. 2024 Jan 15;17(5):1677–94. doi: 10.1039/d3ee02814c (PMC10913155; doi:10.1039/d3ee02814c)
Supplement: EE-017-D3EE02814C-s001 [file EE-017-D3EE02814C-s001.pdf]

Electronic Supplementary Material (ESI) for Energy & Environmental Science.
This journal is © The Royal Society of Chemistry 2024

Supplementary Information for:
Comparing the net-energy balance of standalone photovoltaic-coupled electrolysis and photoelectrochemical hydrogen production

Brian Tam,[1,2] Oytun Babacan,[3] Andreas Kafizas,[2,3] and Jenny Nelson,[1,3]
1 Department of Physics, Imperial College London, South Kensington, London SW7 2AZ, UK
2 Department of Chemistry, Molecular Sciences Research Hub, Imperial College London, White City, London W12 0BZ, UK
3 Grantham Institute - Climate Change and the Environment, Imperial College London, South Kensington, London SW7 2AZ, UK
*Correspondence: b.tam18@imperial.ac.uk (B.T.), jenny.nelson@imperial.ac.uk (J.N.)

S.1 Vensim model and calculations for the ERoEI of a PV-E facility

The diagram of the model shown in Figure S1 was constructed in the system modelling software Vensim PLE+. The arrows show which "variables" are involved with other variable parameters. Variables are recalculated with each iteration if any arrows are pointing into them. They are static if arrows are only pointing out from them. "Levels" are parameters summed over iterative time and are boxed in the diagram.

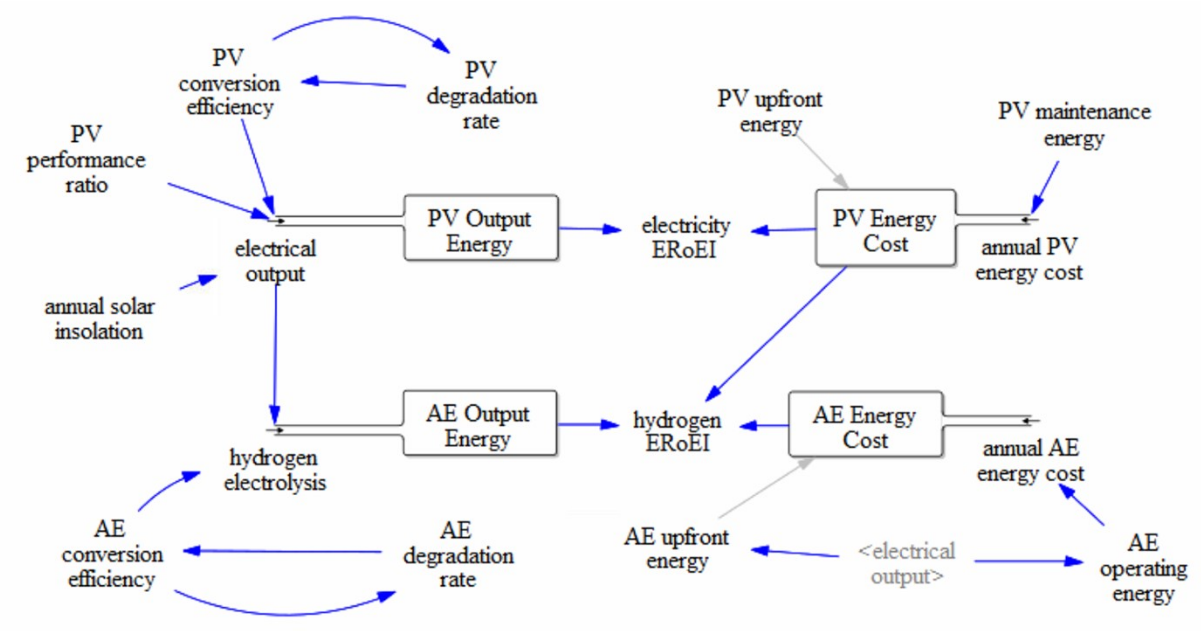

Figure S1. The model diagram for simulating the ERoEI over time for a PV-AE system is recalculated iteratively every year. Blue arrows point to where each variable participates in a calculation. Grey arrows point to where a parameter is involved as an initial condition.

Equation S1 shows the hydrogen energy output as the sum over time 0 to the end of year n of the AE electricity-to-H_2 conversion efficiency at that year, μ_{AE}(year) multiplied by the annual PV solar-to-electricity energy output, $\left(E_{out}^{PVelectricity}\right)_n$. The AE conversion efficiency, detailed in Equation (3), is set to reduce by a degradation rate, (D_{AE}), of its value each year. The conversion efficiency at year 0 is set to an initial value, $\mu_{AE}(0)$.

$$\left[E_{out}^{H_2}\right]_n = \sum_0^n \left[\mu_{AE}(n) \times \left(E_{out}^{PVelectricity}\right)_n\right] \quad \text{(S1)}$$

$$\mu_{AE}(n) = \mu_{AE}(n-1) - D_{AE} \times \mu_{AE}(n-1) \quad \text{(S2)}$$

The annual electrical energy per meter squared of PV active area produced by the PV facility is shown in Equation (S3). It is a function of three parameters: PV solar to electricity conversion efficiency at that year, $\mu_{PV}(n)$, PV performance ratio, PR_{PV},[1] and solar insolation, S, in kWh (m^{-2} of PV) $year^{-1}$.

$$\left[E_{out}^{PVelectricity}\right]_n = \mu_{PV}(n) \times PR_{PV} \times S \quad \text{(S3)}$$

$$\mu_{PV}(n) = \mu_{PV}(n-1) - D_{PV} \times \mu_{PV}(n-1) \quad \text{(S4)}$$

For simplicity, the PV conversion efficiency, shown in Equation (S4), is also assumed to reduce by a degradation rate, D_{PV}, of its value each year. The conversion efficiency at year 0 is also set to an initial value, $\mu_{PV}(0)$.

The cumulative primary energy cost to the system per meter squared of PV, detailed in Equation (S5), is the sum of the upfront energy cost needed to construct the facility, $\left(E_{in}^{construc.}\right)$, the cumulative operating energy cost of the facility from time 0 to the end of year n, $\left[E_{in}^{opera.}\right]_n$, and the anticipated energy cost associated with decommissioning the facility at the end of its operating lifetime, $\left(E_{in}^{decom.}\right)$.

$$\left[E_{in}^{lifecycle}\right]_n = \left(E_{in}^{construc.}\right) + \left[E_{in}^{oper.}\right]_n + \left(E_{in}^{decom.}\right) \quad \text{(S5)}$$

The construction energy cost, shown in Equation (S6), is the sum of the upfront energy embodied in the active components, $\left(E_{in}^{active}\right)$, and in the balance of systems (BOS) i.e. frames, wiring, and gas handling, $\left(E_{in}^{BOS}\right)$. The energy cost of the electrolyser is calculated per meter square of PV active area.

$$\left(E^{construc.}_{in}\right) = \left(E^{active}_{in}\right) + \left(E^{BOS}_{in}\right) \tag{S6}$$

The ongoing operating energy costs of the PV-E facility is the sum of the annual energy cost of running the facility including for power converters, power handling, gas handling and compression. The energy cost of decommissioning the facility depends on whether the spent materials will be landfilled or recycled into new commodity materials or modules. Using Equations (S1)-(S6), the ERoEI as a function of time for a PV-E facility can be simulated with Equation (S7):

$$\text{ERoEI(n)} = \frac{\left[E^{H_2}_{out}\right]_n}{\left[E^{lifecycle}_{in}\right]_n} = \frac{\sum\left[\mu_{AE}(n) \times \left(E^{PVelectricity}_{out}\right)_n\right]}{\left(E^{construc.}_{in}\right) + \left[E^{oper.}_{in}\right]_n + \left(E^{decom.}_{in}\right)} =$$

$$\frac{\sum_{0}^{n}\left[\left(\mu_{AE}(n-1) - D_{AE} \times \mu_{AE}(n-1)\right) \times \left(\mu_{PV}(n-1) - D_{PV} \times \mu_{PV}(n-1)\right) \times \right.}{\left(E^{active}_{in}\right) + \left(E^{BOS}_{in}\right) + \left[E^{oper.}_{in}\right]_n + \left(E^{decom.}_{in}\right)} \tag{S7}$$

Similarly to the ERoEI of the PV-E facility, an ERoEI for a PV facility alone may be calculated for electricity output divided by thermal energy inputs as in Equation S8. The numerator may be calculated from the product of the annual insolation, solar-to-electricity conversion rate, and performance ratio, and the denominator is the sum of the energy costs of building, maintaining, and decommissioning the PV facility.

$$ERoEI(n) = \frac{S \times \mu_{PV} \times PR_{PV}}{\left(E^{active}_{in}\right) + \left(E^{BOS}_{in}\right) + \left[E^{oper.}_{in}\right]_n + \left(E^{decom.}_{in}\right)} \tag{S8}$$

S.2 Vensim model and calculations for the ERoEI of a PEC facility

Figure S2 is the diagram of the model for calculating the PEC ERoEI with its associated relationships. The model follows the approach for simulating the PV-AE system above. Equation 2.1 is again used as the starting point. The model diagram has fewer variables due to the relative simplicity of a PEC water splitting device.

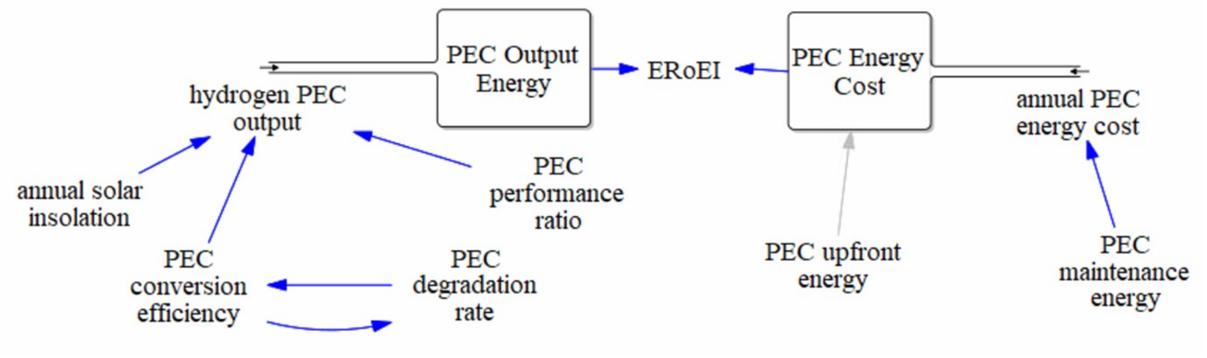

Figure S2. The model diagram for calculating the ERoEI of a PEC system with annual iteration

Equation S9 calculates the PEC annual energy output. The PEC conversion efficiency, shown in Equation (S10), is set to reduce by a degradation rate (D_{PEC}) of its conversion efficiency each year. The conversion efficiency at year 0 is defined as an initial value, $\mu_{PEC}(0)$.

$$\left[E_{out}^{H_2}\right]_n = \mu_{PEC}(n) \times PR_{PEC} \times S \tag{S9}$$

$$\mu_{PEC}(n) = \mu_{PEC}(n-1) - D_{PEC} \times \mu_{PEC}(n-1) \tag{S10}$$

The energy input cost per meter squared of light collection area, detailed in Equation (S11), is the sum of embodied energy cost needed to construct the facility, the cumulative operating energy cost of the facility, and the energy cost associated with decommissioning the facility at the end of its operating lifetime.

$$\left[E_{in}^{lifecycle.PEC}\right]_n = \left(E_{in}^{construc.PEC}\right) + \left[E_{in}^{oper.PEC}\right]_n + \left(E_{in}^{decom.PEC}\right) \tag{S11}$$

The energy cost of constructing the facility, shown in Equation (S12), is the sum of the upfront, embodied energy in the active components of the PEC modules and in the BOS.

$$\left(E_{in}^{construc.PEC}\right) = \left(E_{in}^{active.PEC}\right) + \left(E_{in}^{BOS.PEC}\right) \tag{S12}$$

Finally, using Equations (S9) – (S12), the ERoEI as a function of time for a model PEC facility can be expressed as Equation (S13):

$$\text{ERoEI}(n) = \frac{\left[E_{out}^{H_2}\right]_n}{\left[E_{in}^{lifecycle.PEC}\right]_n} = \frac{\sum\left[S \times PR_{PEC} \times \mu_{PEC}(n)\right]}{\left(E_{in}^{construc.PEC}\right) + \left[E_{in}^{oper.PEC}\right]_n + \left(E_{in}^{decom.PEC}\right)}$$

$$= \frac{\sum_{0}^{n}\left[S \times PR_{PEC} \times \left(\mu PEC(n-1) - D_{PEC} \times \mu PEC(n-1)\right)\right]}{\left(E_{in}^{active.PEC}\right) + \left(E_{in}^{BOS.PEC}\right) + \left[E_{in}^{oper.PEC.annual}\right]_n + \left(E_{in}^{decom.PEC}\right)} \quad (S13)$$

S.3 Full descriptions of the PV-E and PEC simulation input parameter values

PV solar-to-electricity conversion efficiency - The average efficiency of newly installed PV panels in Switzerland in 2015 was 17% after rising by 1% every 2 years from 2005.[2] According to this trend, the average efficiency would be expected to reach **21%** in the present year 2023 and this value is used as the base-case parameter. In fact, commercial panels with advertised efficiency up to 22.8-23.0% are now available from two manufacturers.[3,4] The favourable-case parameter used is the record **26.8%** efficiency for a single-junction silicon-based solar cell with an area of 274 cm^2 [5] tabulated in the latest Solar cell efficiency tables (version 62).[6] It should be noted that multi-junction solar cells involving silicon can achieve higher efficiencies and are being developed at commercial-scale for perovskite/Si cells,[6] which may be important to consider in the future if they ultimately take-up market share from single-junction cells. The optimistic future-case value is taken as approximately **29%** which approaches the theoretical limits calculated by several sources for single-junction cells,[7–9] and may be conservative for future multi-junction solar cells.

PV efficiency degradation – The base-case parameter used comes from the latest (4th) edition of the Methodology Guidelines on Life Cycle Assessment of Photovoltaic Electricity[10] which recommends a degradation rate of **0.7%** for mature module technologies. This rate agrees with the 0.67% recommended by the International Energy Agency Photovoltaic Power Systems Programme (IEA PVPS) 2011 task 12 guidelines assuming linear power degradation of 20% over 30 years.[11] Furthermore, a degradation rate of **0.5%** is recommended for use in sensitivity analyses,[10] which is used here in the favourable case. For the optimistic-case parameter, a value of **0.3%** is taken from the approximate median degradation rates among recent silicon-based devices in a pair of meta-analyses on PV degradation.[12,13] These sources showed a wide range in reported degradation, so the optimistic-case parameter reflects optimal environmental factors rather than an estimate of future technological advancement.

PV performance ratio - The base-case and favourable-case parameters used comes from the latest (4th) edition of the Methodology Guidelines on Life Cycle Assessment of Photovoltaic Electricity[10] which reports that typical performance ratios today are **80% - 90%**, according to the Fraunhofer Institute for Solar Energy Systems latest Photovoltaics Report.[14] These values are a moderate improvement over the values of 70-80% found in a review of life cycle analyses of single-crystalline and multi-crystalline silicon PV systems between 1995 and 2014,[1] and are in agreement with the 0.80 used for ground-mounted utility PV installations.[15,16] Although direct PV-coupled electrolysis facility may not require DC-AC conversion, a PV module under optimal conditions may still need a DC-DC converter to ensure the optimal voltage is supplied; this tends to result in a 5-10% power loss,[17] so **95%** is chosen as the optimistic-case bound.

PV upfront energy cost – A few sources report the embodied energy cost for constructing PV facilities. In 2013, de Wild-Scholten reported a total primary energy demand for multicrystalline silicon PV systems of 2,661.5 MJ m^{-2} (739 kWh m^{-2}) using the electricity mix in China.[18] Meanwhile, Goerig & Breyer reported a market-weighted average primary energy intensity of 3.8 GJ m^{-2} (1056 kWh m^{-2}) for ground-mounted systems and 2.7 GJ m^{-2} (750 kWh m^{-2}) for rooftop systems between 1974-2010.[19] Later, Raugei *et al.* calculated a primary embodied energy cost of 2.76 GJ.m^{-2} (767 kWh.m^{-2}) based on Goerig's work considering 95 % rooftop and 5 % ground-mounted systems in Switzerland.[2] 750 kWh m^{-2} is used in this work as an approximate value for the construction energy for ground-mounted systems, given the limited availability of sources. The energy for decommissioning a PV facility is seldom reported[20] nor even tracked.[21] Therefore, we use here the approximately 5% energy costs reported for landfilling CdTe thin film modules without recycling.[22] Using this value and adding decommissioning costs to construction energy cost yields a final upfront embodied energy cost in the base case of **788 kWh m^{-2}.**

An embodied energy of **537 kWh m^{-2}** is used in this work as the favourable-case present parameter calculated by applying a learning rate of 12% for every doubling of cumulative installed capacity,[23] which agrees with another report of 8-11% and 11-14% for ground-mounted facilities and rooftop systems respectively.[19] The IEA's 2023 Snapshot of Global PV Markets report shows that between 2013 and 2022, cumulative PV installations rose from 137 to 1185 GWp,[21] or roughly three doublings. Applying a 12% reduction to the base-case embodied energy three times results in the favourable-case value. (We assume

here that these trends are suitable for the silicon-based PV models because silicon-based PV makes up a large majority of the global PV market.)

The optimistic future-case value for the embodied energy is calculated as **322 kWh m^{-2}** assuming a moderate slowing in the growth rate resulting in a further four doublings of cumulative installed capacity in the next 20 years. Although this assumption is a rough estimate and ambitious compared to the projections made in a 2019 IRENA analysis,[24] the realised installed PV capacity to 2022 is already ~10% larger[21] than the IRENA projections and the learning curves in past IPCC reports were recently shown by Way *et al.*[25] to consistently overestimate costs and underestimate improvements.

PV maintenance energy cost – To account for the energy costs for replacing faulty equipment, the 2017 analysis by Raugei *et al.* use 1% of the total embodied energy cost as an optimistic bound.[2] Specifically, to calculate this value they note a decrease in the incidence of equipment failure by 86% over the decade up until 2013 reported by the company TUV Rheinland.[2] Primary literature is, however, difficult to source and other reports on technical risks note clearly that PV inverter failure rates are rarely disclosed by manufacturers.[26] One 2020 technical report from NREL for modelling the financial operation and maintenance costs of photovoltaic systems uses an inverter cost of 0.74% of the embodied energy cost for a 10 MW ground-mounted system.[27] We conservatively chose a PV maintenance energy costs of 1% of the PV embodied energy costs in each parameters case. In the base case, the maintenance energy cost is **7.9 kWh m^{-2}** and the favourable and optimistic future-case values are **5.4 kWh m^{-2}** and **3.2 kWh m^{-2}** respectively.

AE conversion efficiency – The base-case and favourable-case values are taken as **65%** conversion efficiency from a 2018 IRENA report for alkaline electrolysers[28] and predicted to rise to **68%** in 2025. This progress agrees with predictions in an expert elicitation study from 2017 that stated system efficiencies would reach 60 to 65% in 2020.[29] A separate 2020 IRENA report also uses 65% for present average conditions, and **76%** for future conditions in 2050 which is taken as the optimistic-case value.[30]

AE efficiency degradation – There are few specific reports of electrolyser efficiency degradation. Most sources instead state a lifetime between 60,000 to 100,000 hours,[30,28] but without knowledge of the typical operation of electrolysers, an annual degradation rate cannot be extracted. Degradation of electrolyser efficiency was reported in one 2015 study[31] for eleven commercial alkaline electrolysers. Specifically, the efficiency degradation was

reported as 1.50% for 2 systems, 1.00% for 4 systems, 0.50% for 2 systems, 0.25% for 2 systems and 0.10% for 1 system. Reviews on alkaline water electrolyzers published in 2018,[32] 2019,[33] and 2021[34] have since cited these values. For this work, the parameters will reflect the variation in the reported data, rather than estimates of future improvement. The base-case value is chosen as **1.50%** annual degradation whereas the favourable-case is **1.00%** degradation and **0.25%** annual degradation is used as the optimistic-case value.

AE upfront energy cost - Pellow *et al.* noted in a 2015 analysis that no peer-reviewed life cycle inventories (LCI) of alkaline water electrolysers were available and so used an empirical LCI of an alkaline fuel cell from 2010 along with Ecoinvent data to estimate an energy cost of 1.36 x 10^6 MJ per MW of capacity for the cell stack (active components).[35] (Since then, life cycle assessments of electrolysers have been published,[36] but do not report energy cost in the form needed here.) Pellow *et al.* also considered a representative commercial gas compressor (RIX Industries model 4VX-S) with an energy intensity of 2.3 x 10^5 MJ per MW. The upfront energy cost of the remainder of the balance of systems was 11.0 x 10^5 MJ per MW by approximating the entire facility as being made from steel. The total energy intensity is finally 2.69 x 10^6 MJ per MW (thermal/electrical).[35] Apart from this report, only one other net-energy analysis by Yadav *et al.* calculate a different energy intensity of 2.79 x 10^6 MJ per MW,[37] after also considering the energy cost of gas compression from Pellow *et al.*[35] A 2019 energy return on investment comparison by Clerjon *et al.*[38] and a 2021 net energy analysis by Lee *et al.* [39] solely use the values from Pellow's work.

Although the total annual solar insolation used for the energy input in this work is 1700 kWh m^{-2} $year^{-1}$, the dimensions of the AE component are sized to match the maximum output from the PV component and lead to an overall capacity factor of ~20% for the electrolyser as was studied by Shaner *et al.*[40] A 1 m^2 PV illuminated with 1700 kWh m^{-2} $year^{-1}$ would have an average power input of 1.94 x 10^{-4} MW. For the base-case parameters, after factoring a 21% conversion efficiency and 0.80 performance ratio, the output power would be 3.26 x 10^{-5} MW. Sizing the AE with an overall capacity factor of 20% then leads to the requirement for an AE that can accept 1.63 x 10^{-4} MW of capacity. Therefore, using Pellow's metric of 2.69 x 10^6 MJ per MW, an AE with embodied energy of 438 MJ m^{-2} of PV is needed, or 122 kWh m^{-2} of PV. While the energy cost of decommissioning alkaline electrolysers is not available, we approximate the costs using 10% of the energy for construction, consistent with multiple energy life cycle assessments for decommissioning natural gas electricity plants done

by the National Energy Technology Laboratory in the United Stated,[41] leading to a final embodied energy of **134 kWh m^{-2} of PV**, for the base-case parameter as a conservative value based on the 2015 Pellow *et al.* analysis .

Although development in electrolyser energy inputs is rarely studied, a 2021 Hydrogen Council report on development of electrolyser monetary costs is available and will be used to estimate a learning curve for the embodied energy inputs.[42] While a 12% learning rate is expected and used in this work, higher rates of 15-20% may also be feasible considering the early development of other technologies such as batteries, solar PV and wind energy.[42] A 2020 IRENA report showing historical water electrolyser capacity between 2005 to 2019 and predictions to 2030 shows that water electrolysis doubled in capacity approximately four times between 2015 and 2023 and may double in capacity another eight times in the next 20 years.[30] Therefore, the base-case energy intensity of 2.79 x 10^6 MJ per MW is reduced to 1.67 x 10^6 MJ per MW and 0.60 x 10^6 MJ per MW in the favourable and optimistic future cases respectively.

Finally, considering the available electricity from the PV modules, in the favourable performance case, with conversion efficiency of 26.8% and 0.90 performance ratio, the power output would be 4.68 x 10^{-5} MW and an AE with 2.34 x 10^{-4} MW of capacity is needed. Therefore, the energy intensity of the AE would be 109 kWh m^{-2} of PV, and the embodied energy including decommissioning would be **119 kWh m^{-2} of PV.** This value will be taken as the favourable case parameter. Similarly, in the optimistic case, the final embodied energy of the AE would be **49 kWh.m^{-2} of PV**.

AE operating energy cost - The operating energy cost of the AE is taken here as largely made up of the energy cost for hydrogen compression. Adiabatic compression of hydrogen to 200 Bar was reported as 12% of the energy of the hydrogen that is being compressed based on its lower heating value (LHV)[3], and as low as 8.5%-9% of hydrogen LHV in a 2020 report based on 2019 findings.[30] Taking an approximate average of value 10% yields an energy cost of **19 kWh m^{-2} year^{-1}** for the base case, considering that insolation of 1700 kWh m^{-2} year^{-1} leads to 286 kWh m^{-2} year^{-1} in electrical output and 186 kWh m^{-2} year^{-1} of hydrogen output.

Future improvements in compression energy costs will likely be physically limited. Therefore, using the average 10% cost of the hydrogen energy produced for the favourable and optimistic-case systems leads to compression energy costs of **28 and 36 kWh m^{-2} year^{-1}** respectively. These values are larger because more hydrogen is produced.

PEC solar-to-hydrogen (STH) conversion efficiency – There are few reports of large-scale PEC water splitting devices in the literature. A 0.4 % STH efficiency was reported by Domen *et al.* for a 1 m^2 particulate photocatalyst device in 2018.[43] An Sb_2Se_3 photocathode and $BiVO_4$ photoanode device with an illuminated area of 0.32 cm^2 showed an overall conversion efficiency of 1.5%.[44] Wired photocatalyst systems often use a PV module to drive water splitting with a single photoelectrode. PV-integrated catalysts for water splitting with area of 64 cm^2 showed a 3.9% STH efficiency.[45] Another PEC-PV device with area of 64 cm^2 showed 4.67% efficiency using a nickel iron molybdenum catalyst.[46] A PEC-PV device with a cobalt-catalyzed tungsten-doped $BiVO_4$ photocatalyst with 0.24 cm^2 illuminated area showed a 5.5-6.3% STH efficiency but these values drop to 1.9-2.1% when the illuminated area rises to 50 cm^2.[47] Higher demonstrated conversion efficiencies for a PEC-PV device, include a 7.1% STH example with 1.5 x 1.5 cm^2 in area,[48] and the record 8.1% STH device from Pihosh *et al.* that reached 90% of the theoretical maximum efficiency for $BiVO_4$ (although for a 4 x 4 mm^2 illumination area).[49] Another $BiVO_4$ tandem device with Cu_2ZnSnS_4 showed a STH of ~8% when coupled to a greenhouse thermoelectric device system.[50]

The Artiphyction project, completed in 2015, yielded the first large-scale 1.6 m^2 PEC prototype using CoPi-catalysed molybdenum-doped $BiVO_4$ which showed initial conversion efficiency of 3% and concluded that further engineering efforts were needed to improve fluid dynamics and to discover better photo-electroactive materials.[51] This value of **3%** conversion efficiency will be chosen as the base-case parameter. **5%** conversion efficiency was their programme target and will be chosen as the favourable-case parameter. Further examples of PEC devices on large-scale demonstration can be found in relevant reviews,[52,53] and at the Solar Fuels Database compiled by EPFL.[54]

The maximum STH efficiency for dual PEC absorbers using high-performance materials is reported to be 28.3% and 16.2% for earth-abundant materials[55] when considering realistic series and shunt resistances and low-performance external radiative efficiencies and catalytic exchange current densities. These values are close to the range of 20-25% that the US DOE is targeting for solar-driven hydrogen production.[56] Predicting near-future conversion efficiency is, however, highly challenging because few PEC devices are in operation and the future materials and configurations of devices may vary greatly from present prototypes. Improvements in large-scale conversion efficiency may occur in leaps and bounds instead of

a gradual climb. **10%** is taken as an illustrative, optimistic future-case conversion efficiency for large-scale PEC devices in the next 20 years, corresponding to, for reference, five doublings of capacity and a 15% learning curve. Further improvement of the conversion efficiency approaching the theoretical limits will likely require additional research and development, although the required timeframe is beyond the scope of this work.

PEC degradation rate (D_{PEC}) – Most studies of experimental PEC devices in the literature only show or test for PEC photoelectrode stability over 1 day or less,[57,58] after which time there is already significant degradation, and even if little to no degradation is observed,[59] annual degradation cannot be reasonably estimated from short experiments. One example of a 1 m^2 SrTiO3:Al panel loaded with cocatalysts was tested for 42 days but showed approximately 40% degradation over this time. Another demonstration of a photoelectrochemical cell was a 50 cm^2 hematite photoanode in tandem with two silicon heterojunction solar cells that reported a very stable performance of 0.04% annualised degradation over 42 days.[60] Upon close inspection, however, there is a drop when considering early plateau regions and performance later on which indicate a **10%** drop in conversion efficiency over the same time. This value will be chosen as the base-case parameter. Other examples of particulate $BiVO_4$ and photoanodes tested in vanadium-saturated electrolyte showed 1000 and 500 hours of stability respectively.[61,62] An annual degradation rate was not reported for the large-scale prototype built by the Artiphyction project, but they do expect to have achieved a lifetime of 10,000 hours, even though operating current decreases by 5 % after the first 300 hours.[51,63]

For the theoretical facilities simulated by Sathre *et al.*,[64] the worst case lifetime of the system is 5 years which corresponds to a **4%** annual linear degradation rate for a facility that reaches the end-of-life when efficiency is reduced by 20% from the initial value. Their base-case lifetime was 10 years, corresponding to a **2%** annual linear degradation rate and these values are chosen here as the favourable-case and optimistic future-case parameters respectively. These metrics are illustrative estimates of the degradation rates of PEC prototypes in the future.

PEC performance ratio (PR_{PEC}) –Similar to PV modules, this ratio for PEC devices includes losses due to shading of the panel from dust and debris, and losses from temperature fluctuations.[1] There is, however, no need to consider losses due to DC to AC conversion or generation and utilisation mismatch so the performance ratio for the PEC device is expected

to be higher than for PV and AE modules. Typical performance ratios for PV modules are 0.75-0.8,[1,11,15,16] reaching 0.835 for multi crystalline PV systems under optimal conditions of high insolation and low ambient temperature.[65] Discounting the losses of 0.1-0.15 to DC to AC conversion, the performance ratio for a PEC module could be expected to reach approximately 0.85-0.95. **85%** will be used as the base-case value and **95%** as the optimistic-case value.

We chose **90%** as an average favourable-case performance ratio, which agrees with an estimate of the performance ratio from the expected energy output. Sathre *et al.*[64] calculates a 31.5 PJ facility^{-1} year^{-1} gross energy output. This output (3.15 x 10^{10} MJ facility^{-1} year^{-1}) for the 4.57 x 10^7 m^2 total module area facility (4.11 x 10^7 m^2 active area) is an energy output density of 689 MJ m^{-2} year^{-1} or 191 kWh m^{-2} year^{-1}. At the very beginning of this simulation, modules operate at 100 % performance instead of 90 %, so the energy output should be calibrated to 213 kWh m^{-2} year^{-1}. A system PR of 0.88 leads to this value for the very initial hydrogen output when applying the annual solar insolation of 2419 kWh m^{-2} year^{-1} and 10% solar-to-hydrogen efficiency used by Sathre *et al*.[64]

PEC upfront embodied energy cost –A theoretical 41.1 km^2 facility with earth-abundant photoelectrodes and precious metal catalysts was predicted by Sathre *et al.* to cost 72.9 PJ (493 kWh m^{-2}) for the initial construction energy cost, (including 981 MJ m^{-2} (272.5 kWh m^{-2}) for the primary energy to manufacture and construct the PEC system without the balance of systems (BOS) and therefore the BOS costs 220 kWh m^{-2}) and 3.4 PJ (23 kWh m^{-2}) for decommissioning[64] for a total embodied energy cost of **516 kWh m^{-2}** and is chosen as the favourable-case performance parameters for this model. Because these parameters are already predictions, no learning curve is applied although the work was published in 2016.

A low-energy cost facility using only earth-abundant photoabsorbers and catalysts is estimated to cost 373 MJ m^{-2} (104 kWh m^{-2}) for the active components.[64] Adding decommissioning and BOS costs assuming that they would be constant whether the active components are energy intensive or not, the low-energy cost scenario has an energy input cost of 104 + 220 + 23 = **347 kWh m^{-2}**. This value is used for the base-case performance parameter. For the optimistic-case future embodied energy cost, a facility similar to the favourable-case system with a mixture of earth-abundant and precious catalysts is assumed to improve over 20 years to an average of the previous cases of **431 kWh m^{-2}**. It is yet to be determined whether low-cost earth abundant PEC catalysts with improved conversion

efficiency or higher-cost precious metal catalysts with lowered costs may ultimately be more effective on an overall net-energy basis in the future.

We note here that unlike for other parameters, the base-case value is smaller than (more favourable than) the favourable-case value because of the variation in the materials assumed. No learning curves are applied to calculating the optimistic future case because of the lack of available data surrounding installed capacity and planning developments.

PEC annual maintenance energy cost – The energy for handling and compressing the gas, the energy for module heating, and for managing water supply was reported by Sathre *et al.* to total an energy cost of 7.3 PJ facility^{-1} year^{-1} (**49 kWh m^{-2} year^{-1}**) for the favourable-case performance system used here.[64] This value is comparable with but larger than the 39.2 kWh m^{-2} year^{-1} effective maintenance cost for the PV-AE system. Proportionally, the base-case and optimistic future-performance case maintenance energy costs are **33 and 41 kWh m^{-2} Year^{-1}** respectively.

S.4 Net-energy balance simulation results

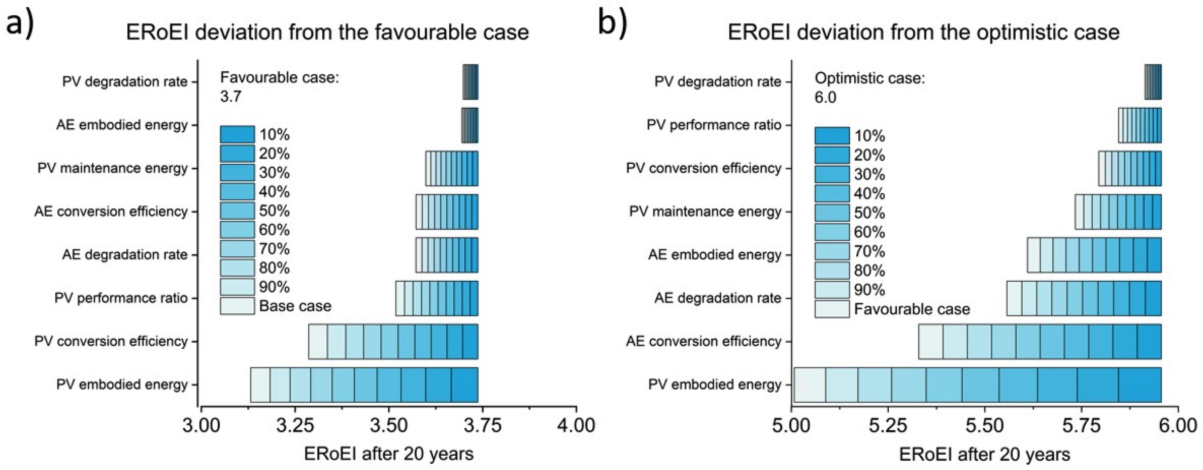

Figure S3. Parameters sensitivity analysis of the ERoEI after 20 years for the PV-E facility for a) varying the individual parameters to their base case in 10% increments while keeping the remaining parameter values at their favourable case estimates, b) varying individual parameters to the favourable case while keeping the remaining parameter values at their optimistic-case values

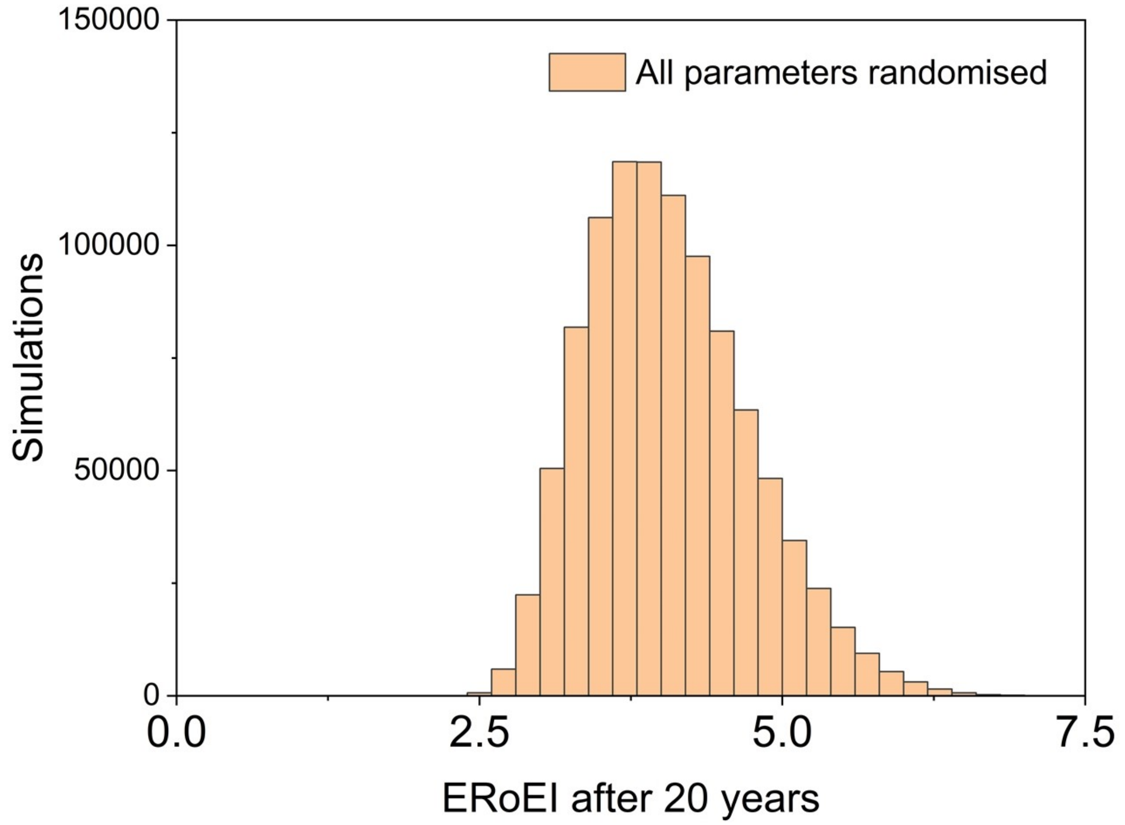

Figure S4. Histogram of Monte Carlo simulation of all parameters of the PV-E facility randomised between their base-case and optimistic-case values.

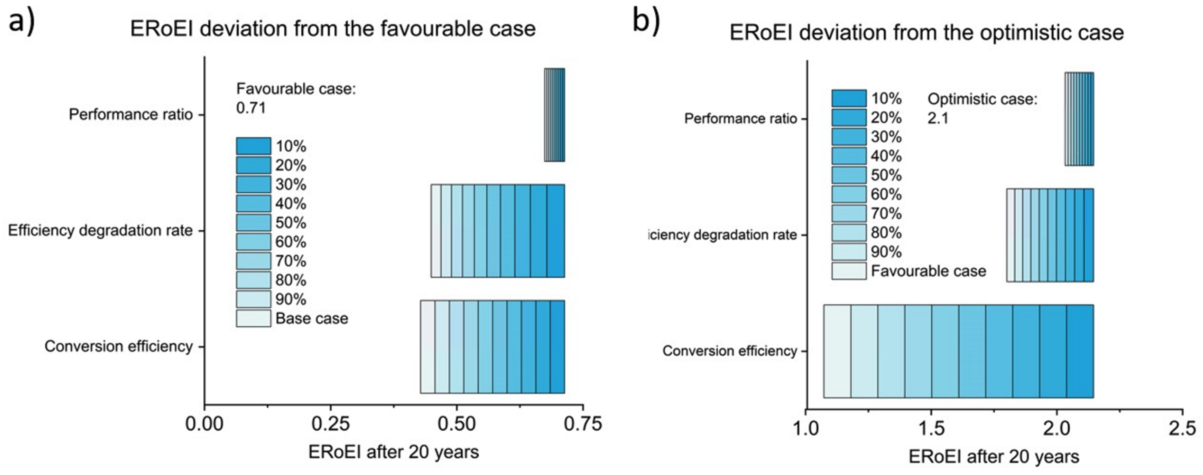

Figure S5. Parameters sensitivity analysis of the ERoEI after 20 years for the PEC facility for a) varying the individual parameters to their base case in 10% increments while keeping the

remaining parameter values at their favourable case estimates, b) varying individual parameters to the favourable case while keeping the remaining parameter values at their optimistic-case values

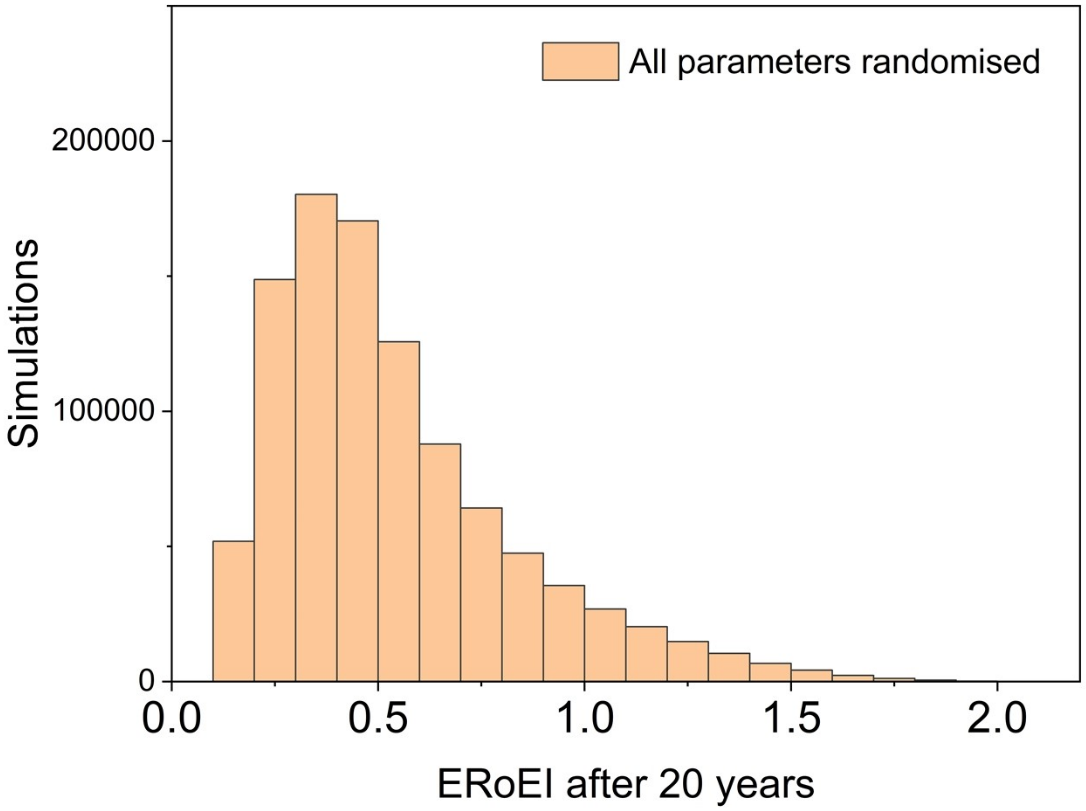

Figure S6. Histogram of Monte Carlo simulation of all parameters of the PEC facility randomised between their base-case and optimistic-case values.

S.5 Recycling energy metrics

Table S1: Embodied energy metrics for each material in a recycled PV-E system.

**Technology**	**Recyclable component materials and % makeup by weight**	**Energy cost to recycle at end-of-life**	**Energy savings when producing recycled materials**<br>**[Energy cost to make the recycled material]**	**Energy cost to produce the new materials, % energy cost portion of the final module**
**Crystalline – Silicon PV**<br><br>Upfront, embodied energy:<br>537 kWh m^{-2}<br><br>Recalculated embodied energy considering recycling (kWh m^{-2}):<br><br>= 7.3 + 1.6 + 0.11 + 0.59 + 306<br>= 316 kWh m^{-2}	**Glass**<br>74.16%[66]<br>65.8%[67]<br>87.3%[68]<br>70%[69]<br>Average ~ 75%	5,634 MJ_{eq} $tonne_{produced}^{-1}$ [70]	6,424 MJ_{eq} $tonne_{produced}^{-1}$ [70]<br>(36.1%)<br><br>[11,371 MJ_{eq} $tonne_{produced}^{-1}$][70]	17,795 MJ_{eq} $tonne_{produced}^{-1}$<br>Derived from [70]
	Net energy – 3%	+ 33% cost	- 36%	1.40%
	**Aluminum** 10.30%[66]<br>17.5%[67]<br>3.53%[68]<br>18.53%[69]<br>Average ~ 15%	5,633 MJ_{eq} $tonne^{-1}$ [70]<br>6-10 MJ kg^{-1} [71]<br>(60-80% recycling efficiency)	187,834 MJ_{eq} $tonne_{produced}^{-1}$ [70]<br>(93.5 %)<br><br>4460 TJ/100,000 Tonnes[71]<br>[240 TJ/100,000 Tonnes][71]	200,892<br>MJ_{eq} $tonne_{produced}^{-1}$<br>Derived from [70]<br><br>4700 TJ/100,000 Tonnes[71]
	Net energy – 90.7%<br>(-89.8%)<br>Average:<br>-90.25%	+ 2.8%	-93.5%[70]<br>(- 94.9%)[71]	3.15%

	**Copper**<br>0.57%[66]<br>1.0%[68]<br>2.14 %[69]<br><br>Average ~ 1%	10,000<br>MJ $tonne_{produced}^{-1}$ [72]<br>6.3 MJ kg^{-1} [71]	90,000 MJ $tonne_{produced}^{-1}$ [72]<br>1060 TJ/100,000 Tonnes[71]	100,000 MJ $tonne_{produced}^{-1}$<br>Derived from [72]
	Net energy – 80%	+ 10%	- 90%	0.10%
	**Silver** 0.004-0.006%[66]<br>0.0013%[68]<br>0.053%[69]<br>Average ~ 0.015%	0.08 MJ m^{-2} [73]<br>For organic photovoltaics	3.4 – MJ m^{-2} [73]<br>For organic photovoltaics	42.17 MJ m^{-2} [73]<br>For organic photovoltaics<br>(0.3048 m^2 per 1.657 g)<br>= 7,757,040 MJ $tonne^{-1}$
	Net energy – 7.9%	+ 0.2%	- 8.1%	0.12%
	**Silicon**<br>3.35%[66]<br>2.9%[67]<br>3.56 %[69]<br>Average ~ 3%	92 kWh_{el}/(12.5 cmx12.5cm)[74]	214 kWh_{el}/(12.5 cmx12.5cm)[74]	306 kWh_{el}/(12.5 cmx12.5cm)[74] assume 1 mm thickness<br>= 30,263,736 MJ $tonne^{-1}$
	Net energy – 40%	+ 30%	- 70%	95.0%
	**Non-recoverable materials (plastics)**<br>5.985%			Energy cost assumed largely recovered by incineration at end-of-life
**Alkaline electrolyser**	**Nickel** catalyst<br>1.9% [75]	1.86 MJ kg^{-1} [71] (186 TJ/100,000 Tonnes)	1878 TJ/100,000 Tonnes[71]	2064 TJ/100,000 Tonnes[71]<br>=20,064 MJ $tonne^{-1}$

Upfront, embodied energy: 119 kWh m^{-2} of PV<br><br>Recalculated embodied energy considering recycling (kWh.m^{-2}):<br><br>0.17 + 32.3 + 5.1 + 21.5 + 0.96 = 60 kWh m^{-2} of PV	Net energy – 82%	+ 9%	- 91%	0.78%
	**Aluminum**<br>7.2% [75]	5,633 MJ$_{eq}$ tonne^{-1} [70]<br>6-10 MJ kg^{-1} [71]<br>(60-80% recycling efficiency)	187,834 MJ$_{eq}$ tonne$_{produced}^{-1}$ [70]<br>(93.5 %)<br><br>4460 TJ/100,000 Tonnes[71]<br>[240 TJ/100,000 Tonnes][71]	200,892<br>MJ$_{eq}$ tonne$_{produced}^{-1}$<br>Derived from [70]<br><br>4700 TJ/100,000 Tonnes[71]
	Net energy – 7.9%	+ 0.2%	- 8.1%	29.5%
	**Copper** wiring<br>10.5% [75]	10,000 MJ.tonne$_{produced}^{-1}$ [72]<br>6.3 MJ kg^{-1} [71]	90,000 MJ tonne$_{produced}^{-1}$ [72]<br>1060 TJ/100,000 Tonnes[71]	100,000 MJ tonne$_{produced}^{-1}$<br>Derived from [72]
	Net energy – 80%	+ 10%	- 90%	21.4%
	**Steel**<br>69.6% [75]	671 kWh = 6357 MJ per tonne[70]	27,176 MJ$_{eq}$ tonne$_{produced}^{-1}$ [70] (81.2 %)	33,468 MJ$_{eq}$ tonne$_{produced}^{-1}$<br>Derived from [70]
	Net energy – 62%	+ 19%	- 81%	47.5%
	**Glass**<br>2.3% [75]	5,634 MJ$_{eq}$ tonne$_{produced}^{-1}$ [70]	6,424 MJ$_{eq}$ tonne$_{produced}^{-1}$ [70]<br>(36.1%)<br><br>[11,371 MJ$_{eq}$ tonne$_{produced}^{-1}$][70]	17,795 MJ$_{eq}$ tonne$_{produced}^{-1}$<br>Derived from [70]
	Net energy – 3%	+ 33% cost	- 36%	0.83%
	**Non-recoverable materials (plastics)**<br>8.6% [75]			Energy cost assumed largely recovered by incineration at end-of-life

Table S2: Embodied energy metrics of each material of a recycled PEC system.

<table>
<tr><th>Technology</th><th>Recyclable component materials and % makeup by weight</th><th>Energy cost to recycle at end-of-life</th><th>Energy savings when producing recycled materials</th><th>Energy cost to make the new materials</th></tr>
<tr><td rowspan="7">Photoelectrochemical water splitting – favourable performance metrics

Upfront, embodied energy:
516 kWh m^{-2}

Recalculated embodied energy considering recycling (kWh m^{-2}):

93 + 113 + 1.9 + 121= 329 kWh m^{-2}</td><td>Glass
207 kg /721 kg (28.7%) total[76]</td><td>5,296 MJ_{eq} per tonne[70]</td><td>6,424 MJ_{eq} $tonne_{produced}^{-1}$ [70]</td><td>17,795 MJ_{eq} $tonne_{produced}^{-1}$ Derived from[70]</td></tr>
<tr><td>Net energy: -3%</td><td>+ 33%</td><td>- 36%</td><td></td></tr>
<tr><td>Steel
340 kg /721 kg (47.2 %) total[76]</td><td>671 kWh = 6357 MJ per tonne[70]</td><td>27,176 MJ_{eq} $tonne_{produced}^{-1}$ [70] (81.2 %)</td><td>33,468 MJ_{eq} $tonne_{produced}^{-1}$ Derived from[70]</td></tr>
<tr><td>Net energy: -62%</td><td>+ 19%</td><td>- 81%</td><td></td></tr>
<tr><td rowspan="2">Light absorbing photocatalysts
4 kg /721 kg (0.55%) total[76]</td><td colspan="2">Materials and modules fabrication based on CdTe</td><td rowspan="2">Materials and modules fabrication 1015 MJ m^{-2} [76]
1190 MJ m^{-2} [22] = 23,471 MJ $tonne^{-1}$</td></tr>
<tr><td>~110 MJ m^{-2} [22]</td><td>~380 MJ m^{-2} [22]</td></tr>
<tr><td>Net energy: -22.7%</td><td>+ 9.2%</td><td>- 31.9%</td><td></td></tr>
</table>

References

1. Wong, J. H., Royapoor, M. & Chan, C. W. Review of life cycle analyses and embodied energy requirements of single-crystalline and multi-crystalline silicon photovoltaic systems. *Renew. Sustain. Energy Rev.* **58**, 608–618 (2016).
2. Raugei, M. *et al.* Energy Return on Energy Invested (ERoEI) for photovoltaic solar systems in regions of moderate insolation: A comprehensive response. *Energy Policy* **102**, 377–384 (2017).
3. SunPower. (2023). Available at: https://go.sunpower.com/affiliate-home-solar/?transaction_id=102216d3a3106af2a2a6c33555f222. (Accessed: 6th November 2023)
4. Products & Solutions. *CanadianSolar* (2023). Available at: https://www.csisolar.com/module/.
5. Lin, H. *et al.* Silicon heterojunction solar cells with up to 26.81% efficiency achieved by

electrically optimized nanocrystalline-silicon hole contact layers. *Nat. Energy* **8**, 789–799 (2023).
6. Green, M. A. *et al.* Solar cell efficiency tables (version 62). *Prog. Photovoltaics Res. Appl.* **31**, 651–663 (2023).
7. Tiedje, T., Yablonovitch, E., Cody, G. D. & Brooks, B. G. Limiting efficiency of silicon intermediate band solar cells. *IEEE Trans. Electron Devices* **31**, 711–716 (1984).
8. Kerr, M. J., Cuevas, A. & Campbell, P. Limiting efficiency of crystalline silicon solar cells due to Coulomb-enhanced Auger recombination. *Prog. Photovoltaics* **11**, 97–104 (2003).
9. Richter, A., Hermle, M. & Glunz, S. W. Reassessment of the limiting efficiency for crystalline silicon solar cells. *IEEE J. Photovoltaics* **3**, 1184–1191 (2013).
10. Frischknecht, R., Stolz, P., Heath, G., Raugei, P. S. & de Wild-Scholten, M. *Methodology Guidelines on Life Cycle Assessment of Photovoltaic 2020*. (2020).
11. Fthenakis, V. *et al. Methodology Guidelines on Life Cycle Assessment of Photovoltaic Electricity, 2nd edition*. (2011).
12. Jordan, D. C. & Kurtz, S. R. Photovoltaic Degradation Rates - an Analytical Review. *Prog. Photovolt Res. Appl.* **21**, 12–29 (2013).
13. Jordan, D. C., Kurtz, S. R., VanSant, K. & Newmiller, J. Compendium of photovoltaic degradation rates. *Prog. Photovoltaics Res. Appl.* **24**, 978–989 (2016).
14. Fraunhofer Institute for Solar Energy Systems. *Photovoltaics Report*. (2022).
15. Fthenakis, V. M., Hyung, C. K. & Alsema, E. Emissions from photovoltaic life cycles. *Environ. Sci. Technol.* **42**, 2168–2174 (2008).
16. Fthenakis, V. & Alsema, E. Photovoltaics Energy Payback Times, Greenhouse Gas Emissions and External Costs: 2004-early 2005 Status. *Prog. Photovolt Res. Appl.* **14**, 275–280 (2006).
17. Bayrak-Pehlivan, I. *et al.* The climatic response of thermally integrated photovoltaic-electrolysis water splitting using Si and CIGS combined with acidic and alkaline electrolysis. *Sustain. Energy Fuels* **4**, 6011–6022 (2020).
18. De Wild-Scholten, M. J. Energy payback time and carbon footprint of commercial photovoltaic systems. *Sol. Energy Mater. Sol. Cells* **119**, 296–305 (2013).
19. Goerig, M. & Breyer, C. Energy Learning Curves of PV Systems. *Environ. Prog. Sustain. Energy* **35**, 914–923 (2016).
20. Windemer, R. Considering time in land use planning: An assessment of end-of-life decision making for commercially managed onshore wind schemes. *Land use policy* **87**, 104024 (2019).
21. International Energy Agency. Snapshot of Global PV Markets 2023 Task 1 Strategic PV Analysis and Outreach PVPS. **I**, 20–45 (2023).
22. Ravikumar, D., Sinha, P., Seager, T. P. & Fraser, M. P. An anticipatory approach to quantify energetics of recycling CdTe photovoltaic systems. *Prog. Photovolt Res. Appl.* **24**, 735–746 (2016).
23. Louwen, A., Van Sark, W. G. J. H. M., Faaij, A. P. C. & Schropp, R. E. I. Re-assessment of net energy production and greenhouse gas emissions avoidance after 40 years of photovoltaics development. *Nat. Commun.* **7**, 1–9 (2016).
24. International Renewable Energy Agency - IRENA. *Future of solar photovoltaic: Deployment, investment, technology, grid integration and socio-economic aspects. A Global Energy Transformation Paper* **November**, (2019).
25. Way, R., Ives, M. C., Mealy, P. & Farmer, J. D. Empirically grounded technology

forecasts and the energy transition. *Joule* **6**, 2057–2082 (2022).
26. Moser, D. *et al.* Technical Risks in PV Projects Development and PV Plant Operation D1.1. D2.1. *Sol. Bankability Proj.* **1**, 1–109 (2016).
27. Walker, A. *et al. Model of Operation-and-Maintenance Costs for Photovoltaic Systems*. *NREL* (2020).
28. IRENA. *Hydrogen From Renewable Power: Technology outlook for the energy transition*. (2018).
29. Schmidt, O. *et al.* Future cost and performance of water electrolysis: An expert elicitation study. *Int. J. Hydrogen Energy* **42**, 30470–30492 (2017).
30. IRENA. *Green Hydrogen Cost Reduction: Scaling up Electrolysers to Meet the 1.5^{0}C Climate Goal*. *International Renewable Energy Agency* (2020).
31. Felgenhauer, M. & Hamacher, T. State-of-the-art of commercial electrolyzers and on-site hydrogen generation for logistic vehicles in South Carolina. *Int. J. Hydrogen Energy* **40**, 2084–2090 (2015).
32. Buttler, A. & Spliethoff, H. Current status of water electrolysis for energy storage, grid balancing and sector coupling via power-to-gas and power-to-liquids: A review. *Renew. Sustain. Energy Rev.* **82**, 2440–2454 (2018).
33. David, M., Ocampo-Martínez, C. & Sánchez-Peña, R. Advances in alkaline water electrolyzers: A review. *J. Energy Storage* **23**, 392–403 (2019).
34. Burton, N. A., Padilla, R. V., Rose, A. & Habibullah, H. Increasing the efficiency of hydrogen production from solar powered water electrolysis. *Renew. Sustain. Energy Rev.* **135**, 110255 (2021).
35. Pellow, M. A., Emmott, C. J. M., Barnhart, C. J. & Benson, S. M. Hydrogen or batteries for grid storage? A net energy analysis. *Energy Environ. Sci.* **8**, 1938–1952 (2015).
36. Zhao, G. *et al.* Life cycle assessment of H_2O electrolysis technologies. *Int. J. Hydrogen Energy* **45**, 23765–23781 (2020).
37. Yadav, D. & Banerjee, R. Net energy and carbon footprint analysis of solar hydrogen production from the high-temperature electrolysis process. *Appl. Energy* **262**, 114503 (2020).
38. Clerjon, A. & Perdu, F. Matching intermittency and electricity storage characteristics through time scale analysis: An energy return on investment comparison. *Energy Environ. Sci.* **12**, 693–705 (2019).
39. Lee, S. *et al.* Comparative energetic studies on liquid organic hydrogen carrier: A net energy analysis. *Renew. Sustain. Energy Rev.* **150**, 111447 (2021).
40. Shaner, M. R., Atwater, H. A., Lewis, N. S. & McFarland, E. W. A comparative technoeconomic analysis of renewable hydrogen production using solar energy. *Energy Environ. Sci.* **9**, 2354–2371 (2016).
41. James III, R. E. *Life Cycle Analysis: Power Studies Compilation Report, National Energy Technology Laboratory Report DOE/NETL-2010/1419*. (2010).
42. Hydrogen Council. *Hydrogen Insights: A perspective on hydrogen investment, market development and cost competitiveness*. (2021).
43. Goto, Y. *et al.* A Particulate Photocatalyst Water-Splitting Panel for Large-Scale Solar Hydrogen Generation. *Joule* **2**, 509–520 (2018).
44. Yang, W. *et al.* Benchmark performance of low-cost Sb2Se3 photocathodes for unassisted solar overall water splitting. *Nat. Commun.* **11**, (2020).
45. Turan, B. *et al.* Upscaling of integrated photoelectrochemical water-splitting devices to large areas. *Nat. Commun.* **7**, 1–9 (2016).

46. Lee, M. *et al.* A Bias-Free, Stand-Alone, and Scalable Photovoltaic–Electrochemical Device for Solar Hydrogen Production. *Adv. Sustain. Syst.* **4**, (2020).
47. Ahmet, I. Y. *et al.* Demonstration of a 50 cm^2 $BiVO_4$ tandem photoelectrochemical-photovoltaic water splitting device. *Sustain. Energy Fuels* **3**, 2366–2379 (2019).
48. Shi, X. *et al.* Unassisted photoelectrochemical water splitting exceeding 7% solar-to-hydrogen conversion efficiency using photon recycling. *Nat. Commun.* **7**, (2016).
49. Pihosh, Y. *et al.* Photocatalytic generation of hydrogen by core-shell WO_3 /$BiVO_4$ nanorods with ultimate water splitting efficiency. *Sci. Rep.* **5**, 11141–11150 (2015).
50. Cai, H. *et al.* Process Accumulated 8% Efficient Cu2ZnSnS4-BiVO4 Tandem Cell for Solar Hydrogen Evolution wiht the Dynamic Balance of Solar Energy Storage and Conversion. *Adv. Sci.* **10**, 2205726 (2023).
51. Tolod, K., Hernández, S. & Russo, N. Recent Advances in the $BiVO_4$ Photocatalyst for Sun-Driven Water Oxidation: Top-Performing Photoanodes and Scale-Up Challenges. *Catalysts* **7**, 13 (2017).
52. Kim, J. H., Hansora, D., Sharma, P., Jang, J. W. & Lee, J. S. Toward practical solar hydrogen production-an artificial photosynthetic leaf-to-farm challenge. *Chem. Soc. Rev.* **48**, 1908–1971 (2019).
53. Moss, B., Babacan, O., Kafizas, A. & Hankin, A. A Review of Inorganic Photoelectrode Developments and Reactor Scale-Up Challenges for Solar Hydrogen Production. *Adv. Energy Mater.* **11**, (2021).
54. SolarFuelsDB. *EPFL* (2022). Available at: https://solarfuelsdb.epfl.ch. (Accessed: 1st September 2022)
55. Fountaine, K. T., Lewerenz, H. J. & Atwater, H. A. Efficiency limits for photoelectrochemical water-splitting. *Nat. Commun.* **7**, 13706 (2016).
56. Hydrogen and Fuel Cell Technologies Office. DOE Technical Targets for Hydrogen Production from Photoelectrochemical Water Splitting. *U.S. Department of Energy* Available at: https://www.energy.gov/eere/fuelcells/doe-technical-targets-hydrogen-production-photoelectrochemical-water-splitting. (Accessed: 19th July 2022)
57. Ager, J. W., Shaner, M. R., Walczak, K. A., Sharp, I. D. & Ardo, S. Experimental demonstrations of spontaneous, solar-driven photoelectrochemical water splitting. *Energy Environ. Sci.* **8**, 2811–2824 (2015).
58. Brillet, J. *et al.* Highly efficient water splitting by a dual-absorber tandem cell. *Nat. Photonics* **6**, 824–828 (2012).
59. Zhou, Y. *et al.* Highly Efficient Photoelectrochemical Water Splitting from Hierarchical WO_3/$BiVO_4$ Nanoporous Sphere Arrays. *Nano Lett.* **17**, 8012–8017 (2017).
60. Vilanova, A., Lopes, T., Spenke, C., Wullenkord, M. & Mendes, A. Optimized photoelectrochemical tandem cell for solar water splitting. *Energy Storage Mater.* **13**, 175–188 (2018).
61. Kuang, Y. *et al.* Ultrastable low-bias water splitting photoanodes via photocorrosion inhibition and in situ catalyst regeneration. *Nat. Energy* **2**, 1–3 (2017).
62. Lee, D. K. & Choi, K. S. Enhancing long-term photostability of $BiVO_4$ photoanodes for solar water splitting by tuning electrolyte composition. *Nat. Energy* **3**, 53–60 (2018).
63. ArtipHyction. *Fully artificial photo- electrochemical device for low temperature hydrogen production*. (2015).
64. Sathre, R. *et al.* Opportunities to improve the net energy performance of photoelectrochemical water-splitting technology. *Energy Environ. Sci.* **9**, 803–819 (2016).

65. Mason, J. E., Fthenakis, V. M., Hansen, T. & Kim, H. C. Energy Pay-Back and Life Cycle CO_2 Emissions of the BOS in an Optimized 3.5 MW PV Installation. *Prog. Photovoltaics* **14**, 179–190 (2006).
66. Paiano, A. Photovoltaic waste assessment in Italy. *Renew. Sustain. Energy Rev.* **41**, 99–112 (2015).
67. Sica, D., Malandrino, O., Supino, S., Testa, M. & Lucchetti, M. C. Management of end-of-life photovoltaic panels as a step towards a circular economy. *Renew. Sustain. Energy Rev.* **82**, 2934–2945 (2018).
68. Goe, M. & Gaustad, G. Strengthening the case for recycling photovoltaics: An energy payback analysis. *Appl. Energy* **120**, 41–48 (2014).
69. Latunussa, C., Mancini, L., Blengini, G., Ardente, F. & Pennington, D. *Analysis of Material Recovery from Silicon Photovoltaic Panels*. *EUR 27797 Luxembourg (Luxembourg) Publications Office of the European Union* (2016). doi:10.2788/786252
70. Rigamonti, L., Grosso, M. & Giugliano, M. Life cycle assessment for optimising the level of separated collection in integrated MSW management systems. *Waste Manag.* **29**, 934–944 (2009).
71. Grimes, S., Donaldson, J. & Cebrian Gomez, G. *Report on the Environmental Benefits of Recycling*. *Bureau of International Recycling* (2008).
72. Copper Development Association Copper Alliance. Copper Recycling and Sustainability. (2018). Available at: https://copperalliance.org.uk/knowledge-base/education/education-resources/copper-recycling-sustainability-2/. (Accessed: 10th November 2021)
73. Søndergaard, R. R., Espinosa, N., Jørgensen, M. & Krebs, F. C. Efficient decommissioning and recycling of polymer solar cells: Justification for use of silver. *Energy Environ. Sci.* **7**, 1006–1012 (2014).
74. Müller, A., Wambach, K. & Alsema, E. Life cycle analysis of solar module recycling process. *Mater. Res. Soc. Symp. Proc.* **895**, 89–94 (2006).
75. Lotrič, A., Sekavčnik, M., Kuštrin, I. & Mori, M. Life-cycle assessment of hydrogen technologies with the focus on EU critical raw materials and end-of-life strategies. *Int. J. Hydrogen Energy* **46**, 10143–10160 (2021).
76. Sathre, R. *et al.* Life-cycle net energy assessment of large-scale hydrogen production via photoelectrochemical water splitting. *Energy Environ. Sci.* **7**, 3264–3278 (2014).
